# Neuroanatomical shifts mirror patterns of ecological divergence in three diverse clades of mimetic butterflies

**DOI:** 10.1111/evo.14547

**Published:** 2022-07-12

**Authors:** J. Benito Wainwright, Stephen H. Montgomery

**Affiliations:** ^1^ School of Biological Sciences University of Bristol Bristol BS8 1TQ United Kingdom

**Keywords:** Brain evolution, Ithomiini, Müllerian mimicry, neuroecology, nonallometric shift, sensory ecology

## Abstract

Microhabitat partitioning in heterogenous environments can support more diverse communities but may expose partitioned species to distinct perceptual challenges. Divergence across microhabitats could therefore lead to local adaptation to contrasting sensory conditions across small spatial scales, but this aspect of community structuring is rarely explored. Diverse communities of ithomiine butterflies provide an example where closely related species partition tropical forests, where shifts in mimetic coloration are tightly associated with shifts in habitat preference. We test the hypothesis that these mimetic and ecological shifts are associated with distinct patterns of sensory neural investment by comparing brain structure across 164 individuals of 16 species from three ithomiine clades. We find distinct brain morphologies between Oleriina and *Hypothyris*, which are mimetically homogenous and occupy a single microhabitat. Oleriina, which occurs in low‐light microhabitats, invests less in visual brain regions than *Hypothyris*, with one notable exception, *Hyposcada anchiala*, the only Oleriina sampled to have converged on mimicry rings found in *Hypothyris*. We also find that *Napeogenes*, which has diversified into a range of mimicry rings, shows intermediate patterns of sensory investment. We identify flight height as a critical factor shaping neuroanatomical diversity, with species that fly higher in the canopy investing more in visual structures. Our work suggests that the sensory ecology of species may be impacted by, and interact with, the ways in which communities of closely related organisms are adaptively assembled.

The diverse ecological opportunities available in heterogenous environments can promote local adaptation, facilitating speciation and the emergence of adaptive radiations (e.g., Rainey and Travisano 1998; Losos 2010). Ecological interactions can have profound evolutionary effects on the species assemblages of entire communities (Johnson and Stinchcombe 2007; Cavender‐Bares et al. 2009), particularly in tropical forest environments where species diversity is high, facilitated by microhabitat partitioning (Willmott and Mallet 2004; Luiselli 2006). Because microhabitats may vary in their sensory information (Chazdon and Fetcher 1984; Endler 1993a; Théry 2001), shifts in ecological preference can expose closely related species to divergent sensory environments, which can lead to adaptive shifts in sensory investment (Powell and Leal 2014; Bloch 2015; Bulova et al. 2016; Scales and Butler 2016; Montgomery and Merrill 2017; Ausprey 2021; Montgomery et al. 2021). There is strong evidence from numerous taxonomic groups that ecological shifts can be accompanied by heritable changes in brain composition, including structures that process sensory information (e.g., Poulson and White 1969; Barton et al. 1995; Catania 2005; Jeffery 2005; Montgomery and Merrill 2017). Patterns of divergence and convergence in sensory traits have received particular attention in radiations of freshwater vertebrates where dramatic environmental gradients have led to rapid local adaptation and diversification at anatomical and molecular levels (Huber et al. 1997; Kotrschal et al. 1998; Sugawara et al. 2005; Hofmann et al. 2009). However, despite their diversity and abundance, adaptive radiations of arthropods have rarely been used as a model system to test similar hypotheses in a terrestrial environment.

Radiations of Neotropical butterflies provide a promising model system for studying patterns of sensory evolution. The Ithomiini (Nymphalidae: Danainae) are a diverse tribe, comprising approximately 400 species across 52 genera, and their ecology, mimetic interactions, and reproductive behaviors have been studied since the birth of evolutionary biology (Bates 1862; Müller 1879; Brown and Freitas 1994; Beccaloni 1997). Across Ithomiini, toxic species converge on similar color patterns, which amplifies their warning signal to predators (Müller 1879; Elias and Joron 2015). Through positive frequency‐dependent selection, this interspecific mutualism has resulted in ithomiines forming the principal models of several Müllerian mimicry rings (also known as mimicry complexes) within sympatric communities. The color pattern diversity of these mimetic species has long puzzled evolutionary biologists (e.g., Bates 1862). However, mounting evidence demonstrates that some mimicry rings are segregated between microhabitats (Poole 1970; Mallet and Gilbert 1995; Beccaloni 1997; DeVries et al. 1999; Elias et al. 2008; Hill 2010; Willmott et al. 2017), suggesting that repeated mimetic shifts could be a significant driver of speciation and ecological segregation in this system. By measuring several abiotic variables and by controlling for phylogeny, Elias et al. (2008) showed that microhabitat segregation of ithomiine mimicry rings occurs across multiple ecological axes, including flight height and topography. Theoretical and empirical studies have confirmed that heterogenous distributions of predators between these microhabitats create local selection pressures, thus maintaining the diversity of mimicry rings observed in nature (Gompert et al. 2011; Willmott et al. 2017). Further to this, agent‐based models have demonstrated that co‐mimetic species assemblages can be maintained through convergence in microhabitat, to enhance warning signal overlap, but not necessarily resource use, enabling niche partitioning within microhabitats (Aubier and Elias 2020). Ithomiine mimicry rings are also vertically stratified by flight height, which is positively correlated with the distribution of their preferred hostplants (Beccaloni 1997). The presence of Müllerian mimicry can therefore predict a species’ ecological niche, permitting the use of color pattern as a proxy indicator of ecological divergence and convergence, and the adaptive assembly of these communities demonstrates that interspecific mutualisms can outweigh the effects of competition and phylogenetic conservatism in shaping ecological niche assemblages (Elias et al. 2008; Hill 2010).

Shifts in mimicry pattern through the colonization of novel microhabitats are therefore likely to play an important role in ecological diversification of ithomiine radiations (Beccaloni 1997; DeVries et al. 1999; Willmott and Mallet 2004; Elias et al. 2009; Chazot et al. 2014). Adaptation to novel sensory cues may arise as a result of exposure to novel environments, or indeed play an active role in facilitating habitat shifts, particularly within complex tropical forests. Indeed, light availability can be extremely variable in forests, posing perceptual challenges for the communities living in them (Chazdon and Fetcher 1984; Endler 1993a; Théry 2001). The well‐documented natural history of ithomiine butterflies, which includes visually guided mating behaviors and hostplant recognition (Pliske 1975; Willmott and Mallet 2004; McClure et al. 2019), makes them a prime case study for exploring adaptations to contrasting visual environments.

Across Lepidoptera, the neuroanatomical structures that process sensory information are well conserved, but the relative volume of individual brain structures, or neuropils, varies hugely between species (Couto et al. 2020). Visual information from the eyes is transmitted via photoreceptor axons to the optic lobe, which consists of the lamina, medulla, lobula plate, lobula, accessory medulla, and, in some species, ventral lobula (Strausfeld and Nässel 1980; Kinoshita et al. 2015). It is within these neuropils where the majority of visual processing takes place (e.g., contrast enhancement, color opponent processing, motion detection), before information is sent to higher integration structures in the central brain such as the mushroom body and anterior optic tubercle, the latter being the most prominent specialized visual neuropil found in the central brain (Morante and Desplan 2004; Borst 2009; Strausfeld 2009; Stӧckl et al. 2020). The relative volume of each neuropil seems to be closely linked to visual ecology. For example, in visually guided species such as *Danaus plexippus*, the optic lobe occupies the majority of the total brain volume (e.g., Heinze and Reppert 2012), whereas in the only ithomiine studied to date, *Godyris zavaleta*, enhanced investment in detecting chemical cues through an enlarged antennal lobe appears to be coincident with a significant reduction of optic lobe total volume, plausibly related to a preference for lower light microhabitats (Montgomery and Ott 2015; Morris et al. 2021). Evidence from *Danaus*, *Godyris*, and other lepidopteran systems (e.g., Montgomery and Merrill 2017; Montgomery et al. 2021) suggests that volumetric investment in sensory structures is positively correlated with the abundance of the relevant sensory cue in a species’ microhabitat. Selection should act on these structures to optimize the execution of behavioral tasks such as hostplant detection, mate signaling, foraging, and predator avoidance.

To test for divergent patterns of neuroanatomical evolution within and between clades, we focus on three ithomiine clades, from two subtribes, which are part of a single, diverse community in eastern Ecuador, the Oleriina, *Napeogenes*, and *Hypothyris* (Fig. 1a). The latter two belong to the subtribe Napeogenina but display a disparity in mimetic diversity. These three clades facilitate comparisons between the mimetically homogenous Oleriina and *Hypothyris*, and the mimetically diverse *Napeogenes*. Levels of local mimetic diversity within all three clades at our study site accurately reflect the diversity observed across these clades as a whole (Chazot et al. 2014; de‐Silva et al. 2016).

**Figure 1:**
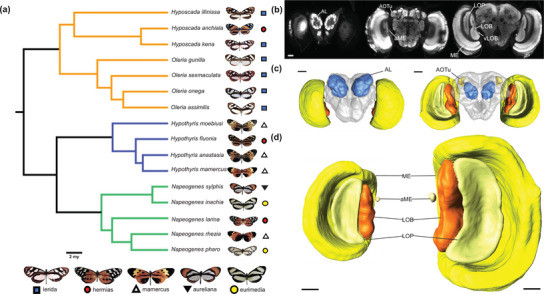
The phylogenetic relationships and neuroanatomical overview of the Ithomiini clades under investigation. (a) A pruned molecular phylogeny calibrated by Chazot et al. (2019), containing all 16 species from our comparative analysis. Colored symbols at the tree tips indicate the mimicry ring to which each species belongs with example models shown at the bottom. Branch colors represent the three monophyletic clades with differing levels of mimetic diversity (orange: Oleriina; blue: *Hypothyris*; turquoise: *Napeogenes*). (b) Anti‐synapsin immunofluorescence from frontal confocal sections of *Hyposcada illinissa* (Oleriina) taken at progressively posterior positions through the brain, moving from left to right. Labeled are the antennal lobes (AL), anterior optic tubercle (AOTu), accessory medulla (aME), medulla (ME), lobula plate (LOP), lobula (LOB), and ventral lobula (vLOB). (c) Dorsal (left) and ventral (right) labeled surface reconstruction of *Hyposcada illinissa* (Oleriina) showing the major sensory neuropil, superimposed on an outline of the rest of the central brain (rCBR) in gray. (d) Posterior surface reconstruction of the primary optic lobe neuropils from *Oleria assimilis* (Oleriina, left) and *Hypothyris anastasia* (*Hypothyris*, right), shown to scale. Scale bars = 100 μm.

The Oleriina and Napeogenina are some of the most speciose subtribes in the Ithomiini, having diversified 15–17 million years ago (de‐Silva et al. 2010, 2016; Chazot et al. 2018). Unlike other ithomiine groups within our study region, species within Oleriina display relative consistency in their mimetic color patterns, with the majority belonging to one mimicry ring (the “lerida” complex) found in inner, dark forests at low elevations (Elias et al. 2008; de‐Silva et al. 2010; Willmott et al. 2017). The majority of species within *Hypothyris* have so‐called “tiger‐stripe” wing color patterns, a phenotype commonly found in more open, sunlit forest or higher in the forest understory (Elias et al. 2008; Willmott et al. 2017). Although there are exceptions (such as *Hyposcada anchiala* that has converged on the tiger‐stripe pattern of *Hypothyris*), the general lack of overlap in mimicry patterns between *Hypothyris* and Oleriina suggests that these two groups may occupy highly divergent sensory niches (Elias et al. 2008; Willmott et al. 2017; Birskis‐Barros et al. 2021). In contrast, within the community sampled, species within *Napeogenes* are segregated into multiple mimicry rings, potentially exposing species to a wider range of sensory stimuli.

Comparative analysis of Oleriina, *Napeogenes*, and *Hypothyris* should therefore provide insights into the evolutionary lability of neuroecological adaptations within adaptive radiations. By focusing on the allometric scaling of the main sensory neuropil of wild butterflies, and by using mimicry as a proxy for habitat preference, we use a comparative approach to reveal how variation in microhabitat diversity may be associated with patterns of adaptation in sensory brain regions. We test for interclade differences between Oleriina and *Hypothyris* to reveal variability in sensory investment between mimetically homogenous clades occupying light and dark forest habitats, and contrast investment within each clade to understand how investment shifts may mirror more recent shifts in microhabitat. Finally, we bolster support that our results reflect ecological patterns of diversity by further testing whether flight height, which is a key aspect of microhabitat partitioning in mimetic butterflies, can explain changes in the scaling of sensory neuropil.

## Methods

### ANIMAL COLLECTION AND DISSECTION

Butterflies were captured using hand nets along designated trails surrounding the Estación Científica Yasuní, in the Parque Nacional Yasuní, Orellana Province, Ecuador where local ithomiine diversity is high (∼60 species). Samples were caught during November/December 2011 and September/October 2012 as part of a larger project to study patterns of sensory evolution across Ithomiini and were caught under permit collection no. 0033‐FAU‐MAE‐DPO‐PNY and exported under permit nos. 001‐FAU‐MAE‐DPO‐PNY and 006‐EXP‐CIEN‐FAU‐DPO‐PNY. These were obtained from Parque Nacional Yasuní, Ministerio Del Ambiente, La Dirección Provincial de Orellana with the help of Estación Científica Yasuní and Pontificia Universidad Católica del Ecuador (PUCE). Focusing on a single community allowed for the control of geographical factors such as altitude and climate and the long‐term nature of the field sampling meant that relative species abundances were representative of the numbers present in the community as a whole. Species were identified using wing venation (Fox 1940) and ID sheets provided by Dr. Keith Willmott for the color pattern races found locally at Yasuní. A total of 16 species were found across the three ithomiine clades of interest (Oleriina, *Napeogenes*, and *Hypothyris*). Sample sizes varied across species from 27 (*Hypothyris anastasia*) to two (*Oleria assimilis*), with an average of 11 per species (Table S1), with the majority having >8 individuals.

Dissections were conducted at the Estación Científica Yasuní under HEPES‐buffered saline (HBS; 150 mM NaCl; 5 mM KCL; 5 mM CaCl_2_; 25 mM sucrose; 10 mM HEPES; pH 7.4). Following Ott (2008), brains were fixed in zinc formaldehyde solution (ZnFA; 0.25% [18.4 mM] ZnCl_2_; 0.788% [135 mM] NaCl; 1.2% [35 mM] sucrose; 1% formaldehyde) for 16–20 h under agitation (for further details, see Montgomery and Ott 2015). Samples were washed three times in HBS and kept in 80% methanol/20% DMSO for at least 2 h under agitation. They were then stored in 100% methanol at room temperature (RT) until they were returned to the United Kingdom where they were transferred to a freezer at −20°C.

### IMMUNOHISTOCHEMISTRY

To reveal neuropil structure, we used indirect immunofluorescence staining against synapsin, a conserved protein found across insects, involved in regulating neurotransmitter release at presynaptic regions (Klagges et al. 1996). Brain samples were rehydrated in a decreasing methanol‐Tris buffer dilution series (90%, 70%, 50%, 30%, and 0%, pH 7.4), each for 10 min. To minimize nonspecific binding, samples were incubated in normal goat serum (NGS; New England BioLabs, Hitchin, Hertfordshire, UK) in 0.1 M phosphate‐buffered saline (PBS; pH 7.4) and 1% DMSO (PBSd) at room temperature for 2 h. Samples were then stained with anti‐SYNORF1 (Antibody 3C11; Developmental Studies Hybridoma Bank, University of Iowa, Iowa City, IA; RRID: AB_2315424) in NGS‐PBSd at a 1:30 dilution for 3.5 days at 4°C under agitation. Any nonbound antibody was removed by conducting three 2‐h washes in PBSd before applying the secondary Cy2‐conjugated anti‐mouse antibody (Jackson ImmunoResearch; Cat No. 115‐225‐146, RRID: AB_2307343, West Grove, PA) at a 1:100 dilution in NGS‐PBSd and incubating at 4°C for a further 2.5 days under agitation. Upon completion, samples were washed in glycerol diluted in 0.1 M Tris buffer (1% DMSO) in a series of increasing concentrations (1%, 2%, and 4% for 2 h each; 8%, 15%, 30%, 50%, 60%, 70%, and 80% for 1 h each) under agitation. They were then dehydrated in 100% ethanol three times, each for 30 min. Finally, to clear the tissue, the ethanol was underlaid with methyl salicylate and left for ∼30 min to allow the brain to sink, before being refreshed with new methyl salicylate.

### CONFOCAL MICROSCOPY

Brains were imaged on a confocal laser‐scanning microscope (Leica SP5‐AOBS/SP5‐II, Leica Microsystem, Mannheim, Germany) at the University of Bristol's Wolfson Bioimaging Facility, using a 10 × 0.4 NA objective lens (Leica Material No. 506285, Leica Microsystem). To image a whole brain, each sample was scanned from the anterior and posterior sides separately using a 488‐nm argon laser (20% intensity) with a mechanical *z*‐step of 2 μm, and an *x*‐*y* resolution of 512 × 512 pixels. Anterior and posterior image stacks were then imported into Amira 3D analysis software 2019.4 (ThermoFisher Scientific, FEI Visualization Sciences Group) where they were merged into a single image stack file using a custom *advanced merge* module provided by Rémi Blanc (Application Engineer at FEI Visualization Sciences Group). To correct for artefactual shortening of the *z*‐dimension after scanning with the 10× objective, the *z* voxel size of the resulting merged stack was multiplied by a factor of 1.52 (as calculated in the analysis of Heinze and Reppert 2012 and Montgomery and Ott 2015).

### IMAGE SEGMENTATION AND VOLUMETRIC RECONSTRUCTION

Image segmentation of the sensory neuropils was also performed in Amira 2019.4. Label files were created for each merged image stack using the *labelfield* module. The boundaries of each neuropil were defined based on the intensity of the synapsin immunofluorescence (Fig. 1b). Every third image through the stack was manually segmented for each neuropil using the paintbrush and magic‐wand tools, before being interpolated in the *z*‐dimension so all intervening unsegmented sections could also be assigned to the neuropil of interest. These interpolated segmentations were then edited in all three dimensions and smoothed before the individual neuropil volumes were extracted using the *measure statistics* module. This procedure was used to reconstruct and measure the volume of five of the six primary neuropils in the optic lobes (medulla, lobula plate, lobula, ventral lobula, accessory medulla) as well as the antennal lobe (the primary olfactory center) and the anterior optic tubercle (AOTu) (the main optic neuropil found in the central brain; Fig. 1c,d). In small ithomiines, the lamina is extremely thin and was damaged during dissections for many samples and was therefore not included. The volume of the medulla, lobula plate, lobula, accessory medulla, and ventral lobula was also summed to calculate a total volume for the optic lobe. The ventral lobula is very small in ithomiines, and in some individuals was apparently absent, with absence being more common among females in most species. Each paired neuropil was measured from one hemisphere only that was chosen at random unless one of the hemispheres was damaged. Once the volume of each paired structure was extracted, it was multiplied by 2 and then log_10_ transformed before any analysis. We also measured the volume of the central brain region and subtracted the raw volume of the antennal lobe and AOTu from this structure (“rest of the central brain” [rCBR]).

### STATISTICAL METHODS

Differences in overall brain composition between clades were visualized by conducting a principal component analysis (PCA) on the dataset. All measured neuropils were included (apart from the ventral lobula due to an abundance of zero values) as well as rCBR. We complemented this with a discriminant function analysis (DFA) to visually illustrate how confidently each individual could be assigned to its respective clade based on volumetric differences in neuropil (using the *MASS* R package, Venables and Ripley 2002).

Differences in neuropil scaling between the three clades were examined by building linear mixed models using the function lmer in the *lme4* package in R (Bates et al. 2015; R Core Team 2016). Each sensory neuropil of interest was scaled against the unsegmented volume from the rCBR that acted as an independent measure of overall brain size and therefore a suitable allometric control. Sex and Species were included as random effects and the effect of Clade was determined by comparing models with and without Clade as a fixed effect using the anova() function. To test for associations between physically and functionally linked neuropils, multiple linear models were also run to build a covariance matrix for all neuropils of interest, the results of which are presented in Table S2E. Ithomiines are not sexually dichromatic, but tests for sexual dimorphism in size were also conducted by running models with Sex as a fixed effect. As we are primarily focused on habitat effects, these results are presented in Table S2C and do not alter the conclusions present in the main text. Species differences in neuropil scaling within clades were also determined by conducting further likelihood ratio tests. In these models, each neuropil was scaled against rCBR with Species as a separate fixed effect and Sex as a random effect. Key comparisons were checked for statistical power using the powerSim function in the *simr* package (Green and MacLeod 2016, see Table S2).

Clade effects may be explained by adaptive processes or be a result of phylogenetic inertia. To better account for these phylogenetic effects, Bayesian phylogenetic mixed models were built using a calibrated and pruned (using *phytools*, Revell 2012) molecular phylogeny from Chazot et al. (2019) (*ape* package; Paradis and Schliep 2018). The R package *MCMCglmm* (Hadfield 2010) controls for phylogenetic nonindependence by including an inverse correlation matrix of the phylogeny as a random effect (inverseA function). A Gaussian distribution was used with uninformative, parameter expanded priors as the random effect (G: *V* = 1, *n* = 1, alpha.*n* = 0, alpha.*V* = 1000; R: *V* = 1, *n* = 0.002) and default priors as fixed effects (MCMCglmm function). Each neuropil was regressed against rCBR with Species included as an additional independent variable and Sex as a random effect. The model was run for 5,000,000 iterations after a burnin of 100,000 for which we report the difference in deviance information criterion (ΔDIC) with and without Species as a fixed effect, where lower DICs indicate a better model fit. DIC is a hierarchical modeling generalization of Akaike's information criterion (AIC) and is the metric offered by the MCMCglmm package where ΔDIC < 2 are considered equivalent. Posterior means, 95% confidence intervals (CIs), and *P*_MCMC_ values for each species are quoted in Table S6D.

If significant clade or species effects were detected in the linear mixed model analysis, post hoc pairwise scaling comparisons were made by constructing standardized major axis regressions (SMATR) using the sma function in the *smatr* package in R (Warton et al. 2012). This tests for differences in the standard scaling relationship (log *y* = *β*log *x* + *α*) by first testing for significant shifts in the slope (*β*), which would reveal an effect of clade or species in the interaction between neuropil and rCBR. If this shift was not found, the presence of a “grade shift” (changes in *α*) was examined, where the allometric slope scaling of each neuropil is the same but vertically shifted along the *y* axis, a common marker of adaptive divergence in brain structure (e.g., Kruska 2005; Sylvester et al. 2011; Montgomery et al. 2016b). If no significant effects were found here, we tested whether scaling differences were due to major axis shifts along a conserved scaling relationship.

Finally, to further explore adaptive hypotheses regarding the observed interspecific differences in sensory brain allometric scaling, we tested the effect of flight height stratification and mimicry pattern. Mean flight height was calculated for each species using raw ecological data from Elias et al. (2008). To overcome low sample sizes and phylogenetically biased distributions of certain mimicry rings, the wing patterns of species were categorized as either being transparent (“aureliana,” “eurimedia,” “lerida”) or colored (“hermias,” “mamercus”) for the mimicry analysis. Using the same priors and model parameters from previous analyses, we ran MCMCglmm models where each neuropil was regressed against rCBR with mean flight height or mimicry included as an additional fixed effect. We report the posterior mean (*P*‐mean) for each species, its 95% CIs, and the probability of the parameter value being different to 0 (*P*_MCMC_).

## Results

### DISTINCT PATTERNS OF SENSORY INVESTMENT BETWEEN MIMETICALLY HOMOGENOUS CLADES SEPARATED ACROSS MICROHABITATS

**Figure 2:**
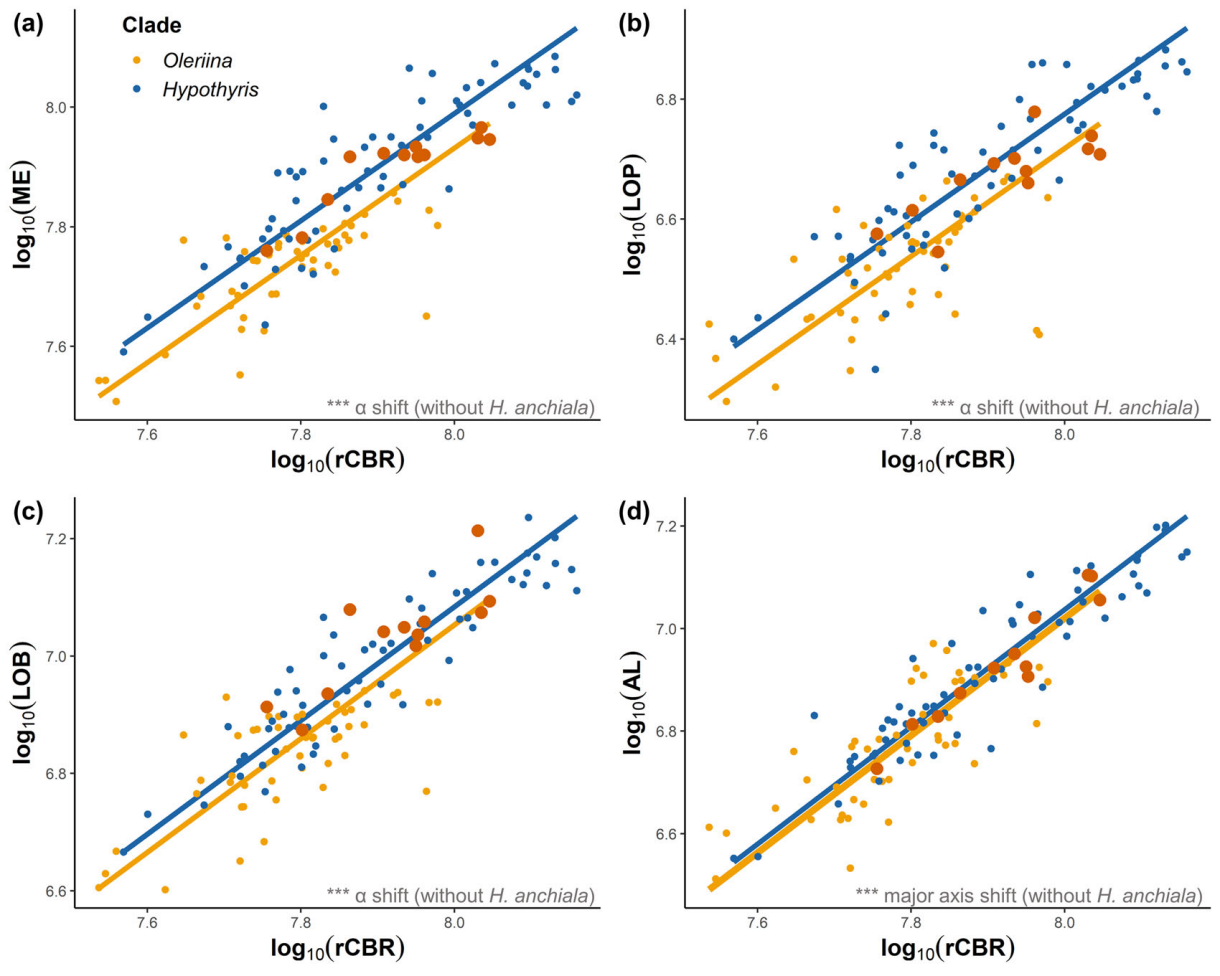
Allometric scaling relationships between the rest of central brain (rCBR) and the medulla (ME) (a), lobula plate (LOP) (b), lobula (LOB) (c), and antennal lobe (AL) (d) of wild‐caught Oleriina and *Hypothyris* individuals, two mimetically homogenous Ithomiini clades. Dark orange data points are from *Hyposcada anchiala*, the only species within the Oleriina clade that does not belong to the “lerida” mimicry ring. Interclade SMATR analysis was run without *H. anchiala* in the dataset. An “*α* shift” denotes a grade shift in the relationship between the two variables, a “*β* shift” denotes a shift in the allometric slope, and a “major axis shift” signifies a main axis shift along a common slope. NS *P* > 0.05, *P < 0.05, **P < 0.01, ***P < 0.001.

**Figure 3:**
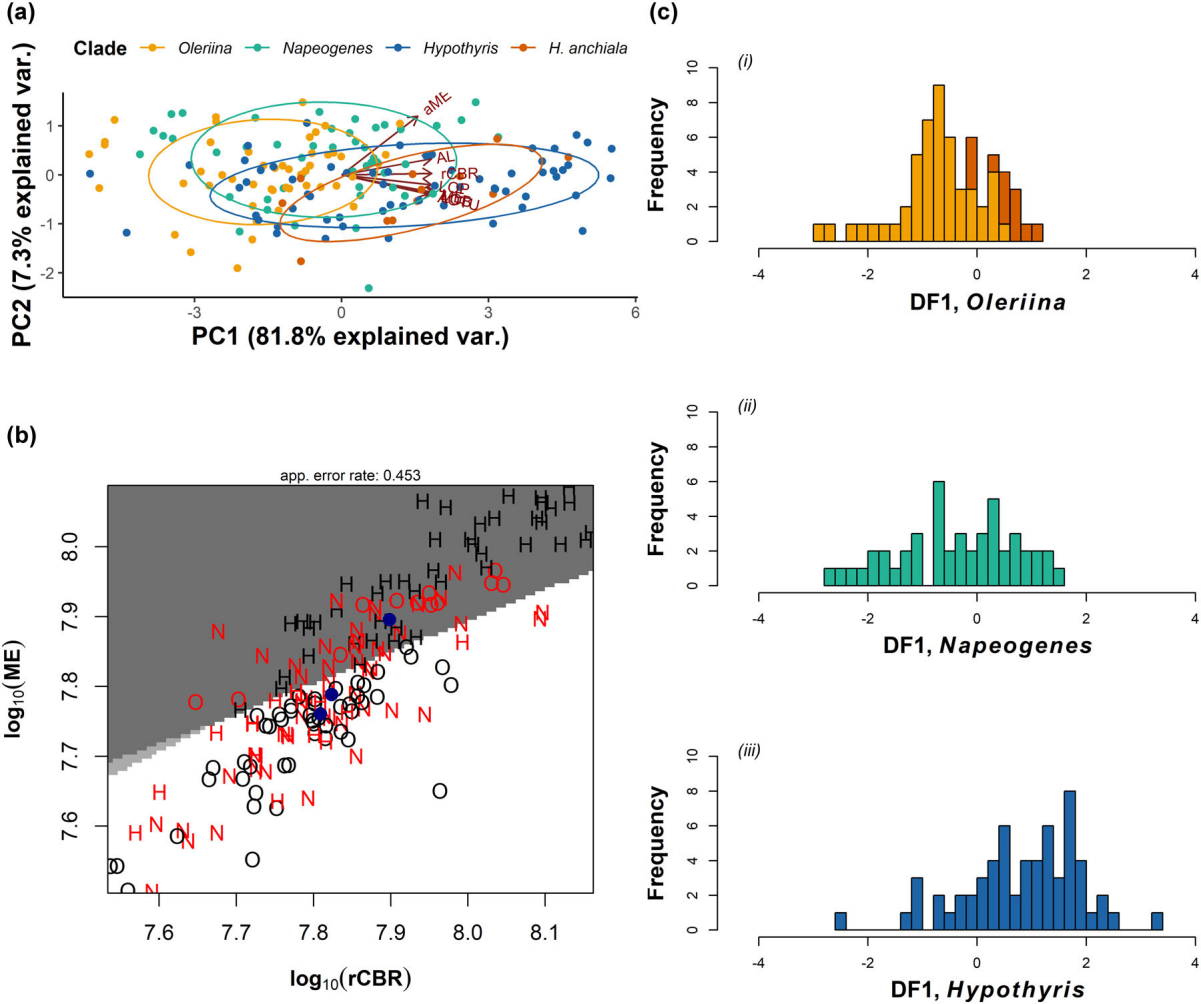
Multivariate analyses of brain morphology between clades. (a) Biplot of PC1 and PC2 from a principal component analysis for all neuropil (apart from the ventral lobula due to an abundance of zero values), in addition to rCBR to control for overall brain size. *Hyposcada anchiala* was included as a separate clade. Vector lengths are proportional to the variance at that neuropil. (b) Partition plot denoting the classification of each individual to its respective clade based on a linear discriminant function analysis for medulla (ME) and rest of central brain (rCBR) total volume. Red signifies an incorrect classification. Means for each clade are plotted in blue. O = Oleriina (dark gray), N = *Napeogenes* (light gray), H = *Hypothyris* (white). (c) Frequency histograms for the observations in each clade (i) Oleriina, (ii) *Napeogenes*, and (iii) *Hypothyris* on the first linear discriminant function. Dark orange bars in (i) represent values for *Hyposcada anchiala*.

When all co‐mimetic species within Oleriina were contrasted with *Hypothyris*, linear mixed models revealed significant clade effects for the scaling of all segmented neuropil, except for the accessory medulla (see Table S2A). SMATR analysis revealed that the majority of interclade differences were a result of grade shifts (changes in the *y*‐intercept) (optic lobe, Wald *χ* ^2^_1_ = 35.78, *P* < 0.001; medulla, Wald *χ* ^2^_1_ = 36.18, *P* < 0.001; lobula plate, Wald *χ* ^2^_1_ = 18.47, *P* < 0.001; lobula, Wald *χ* ^2^_1_ = 12.42, *P* < 0.001; AOTu, Wald *χ* ^2^_1_ = 5.189, *P* = 0.022) with the exception of the ventral lobula and antennal lobe that showed significant slope (Likelihood ratio_1_ = 12.23, *P* < 0.001) and major axis shifts (Wald *χ* ^2^_1_ = 24.620, *P* < 0.001), respectively (Fig. 2). Sexual dimorphism was also observed for the optic lobe as a whole, medulla, ventral lobula, antennal lobe, and AOTu, but effects of the clade and species are independent of sex effects (see Table S2C). The grade shifts demonstrate that, for any given brain size, almost all the visual neuropils are larger in *Hypothyris* than Oleriina (Fig. 2). Although a PCA for all individuals summarized variation in brain structure mostly along a single principal component (PC1) (Fig. 3a; see Table S4A), a DFA on the two clades demonstrated that Oleriina (85.71%) and *Hypothyris* (80.32%) can be discriminated with relative ease based on the size of sensory brain regions (Fig. S1b,c), again indicating the presence of distinct nonallometric variation.

### A SHIFT IN MIMICRY IS ASSOCIATED WITH CONVERGENCE IN BRAIN STRUCTURE BETWEEN *H. anchiala* AND *Hypothyris*

Within Oleriina, there is evidence for interspecific variation in the size of all neuropils except for the antennal lobe, AOTu, and ventral lobula (optic lobe, *χ* ^2^_6_ = 26.167, *P* < 0.001; medulla, *χ* ^2^_6_ = 24.279, *P* < 0.001; lobula plate, *χ* ^2^_6_ = 18.625, *P* = 0.005; lobula, *χ* ^2^_6_ = 29.881, *P* < 0.001; accessory medulla, *χ* ^2^_6_ = 19.125, *P* = 0.004). Notably, when the same analysis was repeated without *H. anchiala*, the only species within Oleriina not belonging to the “lerida” mimicry complex, all species effects become nonsignificant, with the exception of the lobula (*χ* ^2^_5_ = 11.147, *P* = 0.049) and accessory medulla (*χ* ^2^_5_ = 23.644, *P* < 0.001), which is not the case when the analysis is repeated with any other species from Oleriina removed from the dataset (see Table S2B), demonstrating a specific impact of *H. anchiala*.

**Figure 4:**
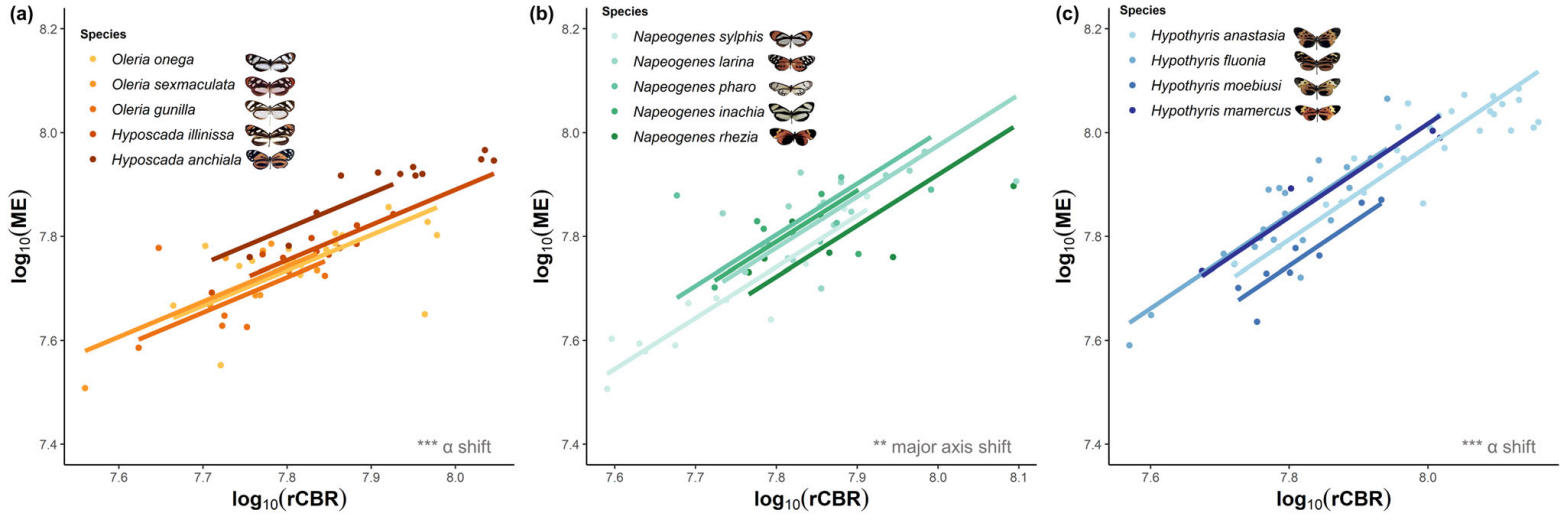
Interspecific scaling relationships between the rest of central brain (rCBR) and the medulla (ME) for Oleriina (a), *Napeogenes* (b), and *Hypothyris* (c). Results from the SMATR analysis are displayed where an “*α* shift” denotes a grade shift in the relationship between the two variables, a “*β* shift” denotes a shift in the allometric slope, and a “major axis shift” signifies a main axis shift along a common slope. NS > 0.05, *P < 0.05, **P < 0.01, ***P < 0.001.

The disproportionate effect of *H. anchiala* is also observed when post hoc SMATR analysis was run on Oleriina for the neuropil that showed significant species effects (Figs. 2, 4, S2a). Significant grade shifts were observed for the optic lobe as a whole (Wald *χ* ^2^_4_ = 41.32, *P* < 0.001), medulla (Wald *χ* ^2^_4_ = 35.09, *P* < 0.001), lobula (Wald *χ* ^2^_4_ = 19.56, *P* < 0.001), and accessory medulla (Wald *χ* ^2^_4_ = 11.77, *P* = 0.019) individually, whereas the lobula plate showed a significant major axis shift (Wald *χ* ^2^_4_ = 52.33, *P* < 0.001). The significant pairwise contrasts were consistently biased toward comparisons with *H. anchiala* (see Table S3C). Therefore, allometric scaling in *H. anchiala* appears to be consistently different across visual neuropils when compared with other species within its clade, which are otherwise generally conserved.

Instead, *H. anchiala* appears to share similar allometry to other “tiger‐stripe” species found within *Hypothyris*, as no significant differences were observed for the scaling of the major visual neuropil when all *Hypothyris* species were contrasted with just *H. anchiala* (see Table S2A). However, despite being a mimetically homogenous clade, significant species effects do still occur within *Hypothyris* for the optic lobe as a whole (*χ* ^2^_3_ = 24.921, *P* < 0.001), medulla (*χ* ^2^_3_ = 24.871, *P* < 0.001), lobula plate (*χ* ^2^_3_ = 20.194, *P* < 0.001), lobula (*χ* ^2^_3_ = 16.462, *P* = 0.001), antennal lobe (*χ* ^2^_3_ = 13.006, *P* = 0.005). and AOTu (*χ* ^2^ = 14.968, *P* = 0.002) (Fig. 4c). Again, the majority of these species effects were the result of grade shifts (see Table S3B). Taken together, these results imply that although *Hypothyris* shows interspecific variation in the relative size of visual brain components, the co‐mimetic *H. anchiala* falls within the range observed in *Hypothyris*, but outside the range of its own clade, the Oleriina, a result clearly inconsistent with the phylogenetic structure of the dataset.

Indeed, across the dataset, the effect of species is also robust to phylogenetic correction using MCMCglmms for all neuropils (optic lobe, ΔDIC = 61.340; medulla, ΔDIC = 61.745; lobula plate, ΔDIC = 33.178; lobula, ΔDIC = 41.911; accessory medulla, ΔDIC = 4.345; ventral lobula, ΔDIC = 14.219; antennal lobe, ΔDIC = 18.133; AOTu, ΔDIC = 8.149). Interestingly, post hoc comparisons from these models show that for the main optic lobe neuropils, when *H. anchiala* was contrasted with all other species, significant differences were found between *H. anchiala* and most other species within its own clade, but not for the majority of co‐mimetic species within *Napeogenes* and *Hypothyris* (see Table S6D). These results provide further evidence that interspecies shifts in neuroanatomy cannot be explained by phylogenetic relatedness alone.

### THE POLYTYPIC GENUS, *Napeogenes*, SHOWS INTERMEDIATE PATTERNS OF INVESTMENT

The output from the PCA and DFA suggests the patterns of allometric scaling for *Napeogenes* to be somewhat intermediate between Oleriina and *Hypothyris* (Fig. 3). Considerable phenotypic overlap with the two mimetically homogenous clades was observed, with only 50% of *Napeogenes* individuals assigned to the correct clade in the DFA, based on neuropil characteristics alone (in contrast to 64% and 70% for Oleriina and *Hypothyris*, respectively) (Fig. 3b,c; see Table S5B). Within *Napeogenes*, significant variation was found across the major visual neuropils, including the optic lobe as a whole (*χ* ^2^_4_ = 17.961, *P* = 0.001), and the medulla (*χ* ^2^_4_ = 18.066, *P* = 0.001), lobula (*χ* ^2^_4_ = 13.491, *P* = 0.009), and ventral lobula (*χ* ^2^_4_ = 11.022, *P* = 0.026) individually. However, upon further inspection, these differences were mostly a result of significant major axis shifts along a common slope (optic lobe, *χ* ^2^_4_ = 15.93, *P* = 0.003; medulla, Wald *χ* ^2^_4_ = 15.81, *P* = 0.003, Fig. 4b; lobula, Wald *χ* ^2^_4_ = 17.75, *P* = 0.001), with the exception of the ventral lobula where a significant *β*‐shift was observed (Likelihood ratio_4_ = 11.10, *P* = 0.025). Pairwise contrasts revealed that all significant comparisons were with *Napeogenes sylphis*. The highly polytypic genus *Napeogenes* therefore appears to be intermediate between more specialized, or mimetically homogenous, clades, with variation in brain structure mainly being explained by variation in overall brain size.

### FLIGHT HEIGHT MAY EXPLAIN CHANGES IN VISUAL INVESTMENT

**Figure 5:**
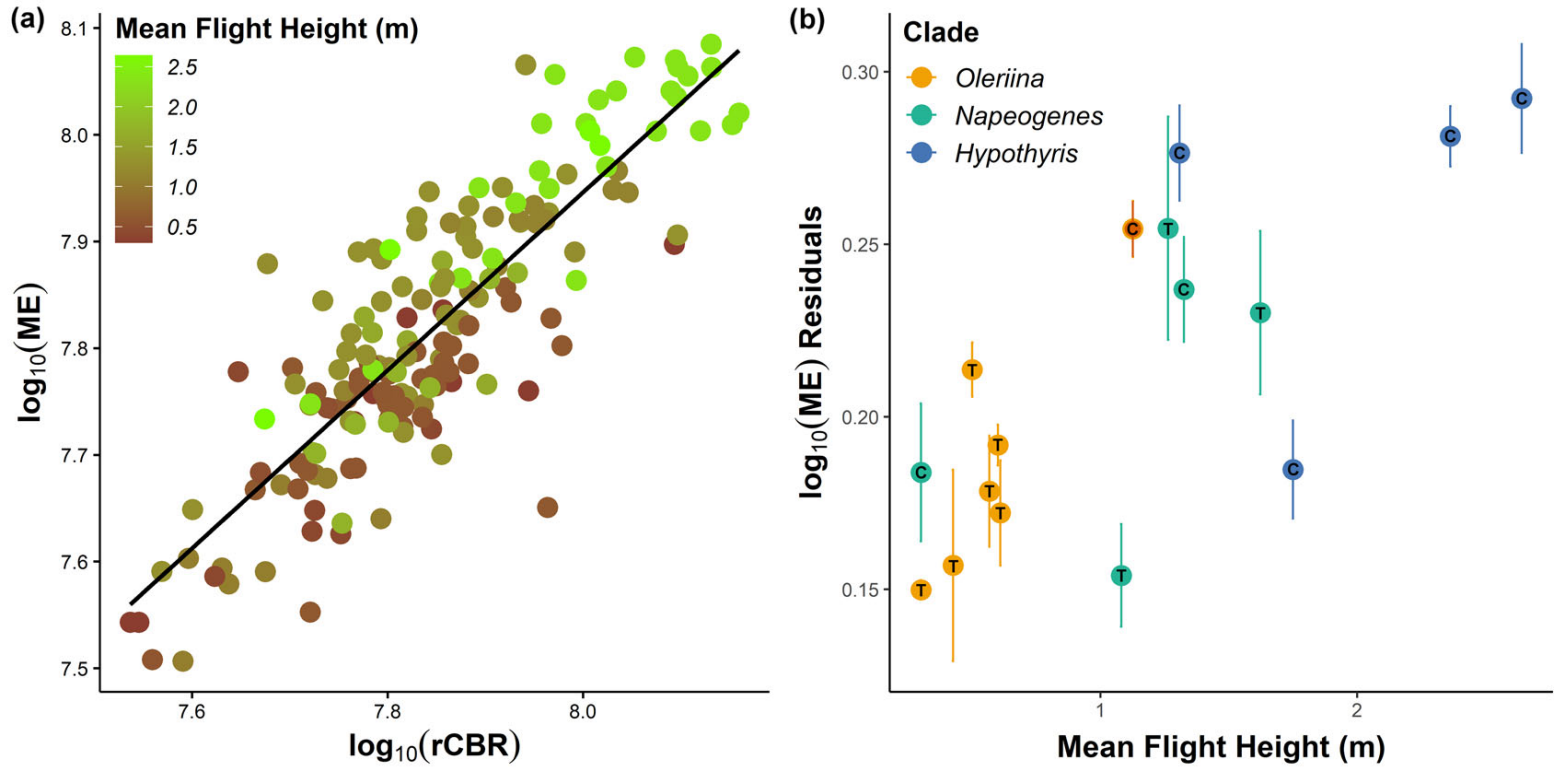
Correlation between flight height (in meters, m) and level of volumetric investment in visual neuropil. (a) The scaling relationship between the medulla (ME) and the rest of central brain (rCBR) for all individuals with different color shades representing the mean flight height for each species, using data from Elias et al. (2008). (b) Residuals from the medulla (ME) for each species, calculated using the regression equation from the MCMCglmm model, plotted against mean flight height (in meters, m). Dark orange point represents *Hyposcada* anchiala, the only species within the Oleriina clade that does not belong to the “lerida” mimicry ring. Letters inside each data point indicate whether species belong to a colored (C) or transparent (T) mimicry ring. Bars indicate standard error in the medulla residual.

The comparisons above are based on comparing clades with differing patterns of mimicry ring diversity as a proxy for diversity in microhabitat preference. We also directly tested whether mimicry ring was a significant predictor of relative sensory investment across all three clades, but these analyses were unable to disentangle effects of mimicry from phylogenetic effects due to the strong phylogenetic clustering of mimicry rings, especially when condensed to a binary trait (transparent/colored) (see Table S6B). However, mimicry ring may also not fully capture ecological preferences of each species. A major contributor to microhabitat partitioning is flight height (Medina et al. 1996; Beccaloni 1997; Willmott et al. 2017), which is associated with exposure to distinct light conditions (Endler 1993a; Matsuo et al. 2021), and could explain some of the variation observed between and within clades. Indeed, after correcting for rest‐of‐brain size and phylogenetic effects, we find significant associations between flight height and the size of the optic lobe as a whole (*P*‐mean = 0.047, 95% CI: 0.008–0.088, *P*_MCMC_ = 0.020), and for the medulla (*P*‐mean = 0.046, 95% CI: 0.006–0.089, *P*_MCMC_ = 0.030, Fig. 5), lobula (*P*‐mean = 0.053, 95% CI: 0.011–0.095, *P*_MCMC_ = 0.015), ventral lobula (*P*‐mean = −1.152, 95% CI: −2.016 to 0.3553, *P*_MCMC_ = 0.005), and AOTu (*P*‐mean = 0.039, 95% CI: 0.002–0.078, *P*_MCMC_ = 0.048) individually. In all these analyses, relative neuropil volume was greatest in species that flew higher in the forest canopy.

## Discussion

In Neotropical butterflies, shifts in mimetic coloration have been shown to reflect divergence in ecological conditions (Beccaloni 1997; Elias et al. 2008; Hill 2010). Here, we present further evidence that these repeated shifts accompany changes in relative sensory investment and hypothesis that these reflect adaptations to divergent visual environments. These results are consistent with similar studies in *Heliconius* where habitat segregation across continuous environmental gradients (Jiggins et al. 1997; Estrada and Jiggins 2002) has been linked to divergence in neuroanatomy and peripheral eye structure (Montgomery and Merrill 2017; Montgomery et al. 2021; Seymoure et al. unpubl. ms.) and sensory biases (Dell'Aglio et al. 2022). Overall, species occupying brighter environments invest relatively more in optic neuropil, likely reflecting the extent of processing performed on locally abundant sensory information. For species living in low‐light conditions, selection might act on other, more peripheral, sensory components that would compensate for low light availability in these microhabitats. For example, nocturnal Lepidoptera have evolved enhanced visual sensitivity by increasing facet and relative eye size (Frederisken and Warrant 2008). Because of its retinotopic nature, such differences might impact the relative size of the lamina (which was not measured in this study), but not necessarily other structures in the optic lobe.

Enhanced species diversity within single communities, where confounding factors such as altitude are of limited importance, makes Ithomiini a powerful model system for studying divergent selection on sensory processing within single rainforest communities. Nonallometric shifts in relative investment, indicated by standardized major axis regressions, strongly suggest that interspecific differences are products of adaptive evolution rather than neutral evolution, which would be more consistent with conserved scaling between species and clades. This is because neural scaling relationships are considered to be a result of stabilizing selection that maintains a functional balance between different brain components (Barton and Harvey 2000; Montgomery and Merrill 2017). Shifts in ecology are likely to accompany selection pressures for changes in functional or neuronal connectivity, optimizing sensory exploitation with minimal energetic costs (Laughlin 2001; Ott and Rogers 2010).

Mimetic homogeneity within Oleriina proved to be an accurate predictor of neuroanatomical similarity. With the exception of *Hyposcada anchiala*, which is part of the tiger‐stripe “hermias” ring, all other species in this clade are transparent co‐mimics within the “lerida” mimicry ring. On the whole, relative investment in visual neuropil was significantly reduced in Oleriina species compared to *Hypothyris* (Fig. 1d) and *Napeogenes*, perhaps reflecting the densely shaded tropical forest in which this community lives, similar to that of *Godyris zavaleta* (Pliske 1975; Hill 2010; Montgomery and Ott 2015). Although this pattern is confounded by phylogenetic relatedness, an enhanced visual investment shift in the “hermias” *H. anchiala* suggests that mutualistic mimetic interactions might outweigh phylogenetic effects in shaping species‐specific sensory ecologies in this subtribe. The grade shift in *H. anchiala* toward expanded visual neuropil is sufficient to match the level of investment found in other “tiger‐stripe” butterflies within *Hypothyris*, which otherwise consistently invest more heavily in visual neuropil than Oleriina (Figs. 2, 4).

Although interclade comparisons between Oleriina and *Hypothyris* highlight the role of microhabitat in predicting patterns of neuroanatomical divergence, several other observations suggest these associations are imperfect. Despite belonging to three separate mimicry rings, the remaining four *Napeogenes* species (other than *N. sylphis*) display little neuroanatomical variation (Fig. 4b). Overall, high mimetic diversity appears to be associated with intermediate neuroanatomical scaling, relative to Oleriina and *Hypothyris*, and within *Napeogenes*, major axis shifts suggest that most interspecific variation can be explained by differences in brain size, rather than structure. It may be that *Napeogenes* is a sensory generalist, able to evolve into more diverse microhabitats by being less specialized to high‐ or low‐light environments. Alternatively, sensory ecological variation between some microhabitats may not be sufficiently different enough to promote significant shifts in visual and olfactory investment between all ithomiine mimicry rings. For example, not all ithomiine mimicry rings differ significantly in flight height or microhabitat type, so the relative light abundance experienced by individual butterflies may not differ between all mimicry rings (Beccaloni 1997; Willmott et al. 2017). This would be analogous to a study conducted in *Anolis* lizards where species from different habitats, but with similar vegetation profiles, displayed similar visual spectral sensitivities (Fleishman et al. 1997). Finally, it is possible the more recent mimetic shifts within *Napeogenes* may not have provided sufficient time for neuroanatomical adaptations to occur, although heritable differences have been reported between even younger *Heliconius* species pairs (Montgomery and Merrill 2017; Montgomery et al. 2021) suggesting neuroanatomical divergence can accumulate over short time periods. Significant grade shifts in visual neuropil scaling within the mimetically homogenous *Hypothyris* are also surprising but interspecific differences in flight height help explain this result (Fig. 5).

The continuous relationship between flight height and visual investment suggests that some interspecific differences within mimicry rings can be explained by other ecological variables. Height stratification of ithomiine mimicry rings is well documented (Beccaloni 1997; Willmott and Mallet 2004; Joron 2005; Willmott et al. 2017), where differences in temperature and humidity across short spatial scales can significantly influence butterfly host‐plant distribution (Checa et al. 2014). As a result, selection for improved foraging and oviposition efficiency constrains species movements between microhabitats, leading to sensory niche specialization that might also be accompanied by changes in intraspecific communication (Endler 1993b). *Hypothyris*, for example, flies higher on average in the forest canopy than the other two clades (Fig. 5), which is likely associated with enhanced light availability at higher elevations through the forest understory (Montgomery and Chazdon 2001) and could explain increased relative investment in their visual neuropil.

Significant clade and flight height associations were also seen for sensory processing neuropils outside of the optic lobes. The AOTu, the main visual neuropil found within the central brain, is known to play an important role in the parallel processing of chromatic and polarization cues in insects, including butterflies (Pfeiffer et al. 2005; Heinze et al. 2013; Mota et al. 2013). These cues might vary between microhabitats and canopy elevations but interestingly, few significant within‐clade differences in allometric scaling were found for the AOTu, suggesting that selection for enhanced visual processing acts more strongly on peripheral structures within the optic lobes (Fig. 2). However, flight height does show a significant association with investment in this structure, perhaps reflecting a richer abundance of chromatic and polarization cues at higher understory elevations. In *Heliconius*, the AOTu shows convergent expansion in species living in closed habitats (Seymoure et al. unpubl. ms.), possibly optimizing the trapline foraging behaviors of these butterflies in habitats where the distribution of resources is sparse. Whether an expanded AOTu provides similar benefits to high flying Ithomiini remains to be seen, but our results suggest that shifts in investment in these regions may only be apparent when comparing between, rather than within, clades.

The ecological shifts we link to divergence in optic lobe investment and structure might also lead to correlated changes in selection regime, or possibly trade‐offs, on other sensory modalities, such as olfaction (e.g., Keesey et al. 2019). Despite significant interclade differences in antennal lobe structure, major axis shifts suggest that these were a result of variation in overall brain size (Fig. 2d), and our within‐clade analysis revealed little evidence of changes in antennal lobe scaling between species. This might be because the odor cues species rely upon are relatively similar between closely related species. Indeed, all the sampled Ithomiini are motivated to forage for pyrrolizidine alkaloid containing plants (Trigo et al. 1996; Morris et al. 2021), and the pheromonal blends used in intraspecific communication are similar between closely related species (Pliske 1975; Schulz et al. 2004). For example, a comparative analysis of male Ithomiini hairpencil compounds (used in both mate attraction and predator defense) revealed that all species within Oleriina lack the plant‐derived pyrrolizidine alkaloids that are otherwise widespread across most of the main ithomiine subtribes (Schulz et al. 2004). A comparison across 13 Ithomiini genera further suggested that this shift in pheromone blend could be associated with a more structurally complex internal antennal lobe morphology within *Oleria* (Morris et al. 2021), which may occur without major volumetric differences in antennal lobe (AL) size. These differences might explain the significant interclade variation in antennal lobe volume from our analyses. To summarize, although mimetic shifts might be associated with changes in visual investment, this may not necessarily result in chemical ecological changes that would promote differential investment in olfactory stimuli.

Our results suggest that shifts in sensory ecology necessitate shifts in investment in sensory processing regions of the brain, but further work is required to better understand the precise functional explanations for these sensory shifts. Although causality is difficult to determine from this study, several lines of evidence suggest that microhabitat shifts are more likely to drive changes in sensory investment than vice versa. First, data in other systems suggest this may be the case, with neural investment closely following patterns of exposure to environmental conditions (Bulova et al. 2016; Musilova et al. 2019). Second, color pattern shifts are thought to be the main trigger of divergence in mimetic butterflies, with ecological divergence accumulating and further facilitating reproductive isolation (Mérot et al. 2017). Finally, in our own data, it is unclear what would drive divergence in brain composition in *H. anchiala* away from the other Oleriina, prior to an ecological shift. Given the wild‐caught nature of our samples, it is also possible these shifts could be explained by phenotypic plasticity. However, common garden experiments in other butterfly systems suggest that differences in developmental plasticity are not sufficient to explain overall species differences in brain composition (Montgomery et al. 2016a, 2021; Montgomery and Merrill 2017).

Our analysis of flight height highlights the importance of precise ecological data for better understanding the factors that shape neuroanatomical diversity, but future studies should also attempt to quantify the light environment for each species’ respective ecology. When collecting ecological data for their phylogenetic comparative analyses, Elias et al. (2008) collected a range of variables, representing forest structure and topography, which were eventually summarized into three principal component axes. However, no direct information on the physical properties of the light or other sensory cues within the different microhabitats was available. Quantifying the sensory environment in the field and modeling it to reflect the sensory capabilities of the study organism is challenging, but detailed knowledge of both butterfly vision (e.g., Briscoe 2008; Arikawa 2017) and ithomiine ecology (e.g., Beccaloni 1997; DeVries et al. 1999; Elias et al. 2008; Hill 2010; Willmott et al. 2017) makes Ithomiini a promising study system for achieving these goals. Carefully controlled experiments are also required that link component volumes to observed behavioral differences. For example, ithomiine species from more sunlit microhabitats might invest more in visual neuropil to optimize mate choice, foraging, hostplant detection, and/or navigation.

In conclusion, communities of ithomiine butterflies provide useful models for studying how sensory ecological adaptations evolve in complex environments. Mutualistic mimetic interactions between species can have profound effects on the evolutionary trajectories of several processing structures within the ithomiine brain. Our work provides one of the most extensive neuroanatomical comparative analyses across any insect order. We have demonstrated that variation in mimetic diversity can often predict shifts in sensory investment, with the primary target of selection being visual processing structures in the optic lobes. These shifts are partly explained by differences in mean flight height, independently of phylogenetic effects. Our study provides hints toward the evolutionary lability of neuroanatomical adaptations within adaptive radiations and their role in ecological divergence between sensory niches in a terrestrial environment. Quantifying differences in light environment characteristics between microhabitats, coupled with larger phylogenetically controlled comparative analysis across a variety of sensory traits, would enhance knowledge into the selective drivers favoring sensory adaptations in a complex tropical environment.

## AUTHOR CONTRIBUTIONS

SHM conceived the study and collected the samples in the field. JBW and SHM designed the analyses. JBW performed the staining, imaging, segmentation, data extraction, and analyses. JBW wrote the first draft of the manuscript, which was subsequently edited by JBW and SHM.

## DATA ARCHIVING

All raw data can be found in Table S1 and are available from Dryad Digital Repository https://doi.org/10.5061/dryad.sj3tx967b (Wainwright and Montgomery 2022).

## CONFLICT OF INTEREST

The authors declare no conflict of interest.

Associate Editor: Dr. J. A. Tobias

Handling Editor: Dr. A. G. McAdam

## Supporting information

Supplementary information

Supplementary information

**Figure S1**: Multivariate analyses of brain morphology between monotypic clades (*Hypothyris* and Oleriina excluding *Hyposcada anchiala*). **(a)** Biplot of PC1 and PC2 from a principal component analyses for all neuropil (apart from the ventral lobula due to an abundance of zero values), in addition to rCBR to control for overall brain size. Vector lengths are proportional to the variance at that neuropil. **(b)** Partition plot denoting the classification of each individual to its respective clade based on a linear discriminant function analyses for medulla (ME) and rest of central brain (rCBR) total volume. Red signifies an incorrect classification. Means for each clade are plotted in blue. O = Oleriina (dark grey), H = *Hypothyris* (white). **(c)** Frequency histograms for the observations in each clade ((i) Oleriina, ii) *Hypothyris*) on the first linear discriminant function.
**Figure S2**: Interspecific scaling relationships between the rest of central brain (rCBR) and the lobula (LOB) for Oleriina **(a)**, *Napeogenes* **(b)**, and *Hypothyris* **(c)**. Results from the SMATR analysis are displayed where an ‘*α* shift’ denotes a grade‐shift in the relationship between the two variables, a ‘*β* shift’ denotes a shift in the allometric slope, and a ‘major axis shift’ signifies a main axis shift along a common slope. NS > 0.05, * P < 0.05, ** P < 0.01, *** P < 0.001

Table S1: Raw Data
Table S2: Linear Mixed Model Output
Table S3: Standardised Major Axis Regression (SMATR) analysis
Table S4: Principal Component Analyses
Table S5: Discriminant Function Analyses
Table S6: MCMCglmm Results
