# Supplementary figures and images for: Neuroanatomical shifts mirror patterns of ecological divergence in three diverse clades of mimetic butterflies
Source: Evolution. 2022 Jul 12;76(8):1806–20. doi: 10.1111/evo.14547 (PMC9540801; doi:10.1111/evo.14547)

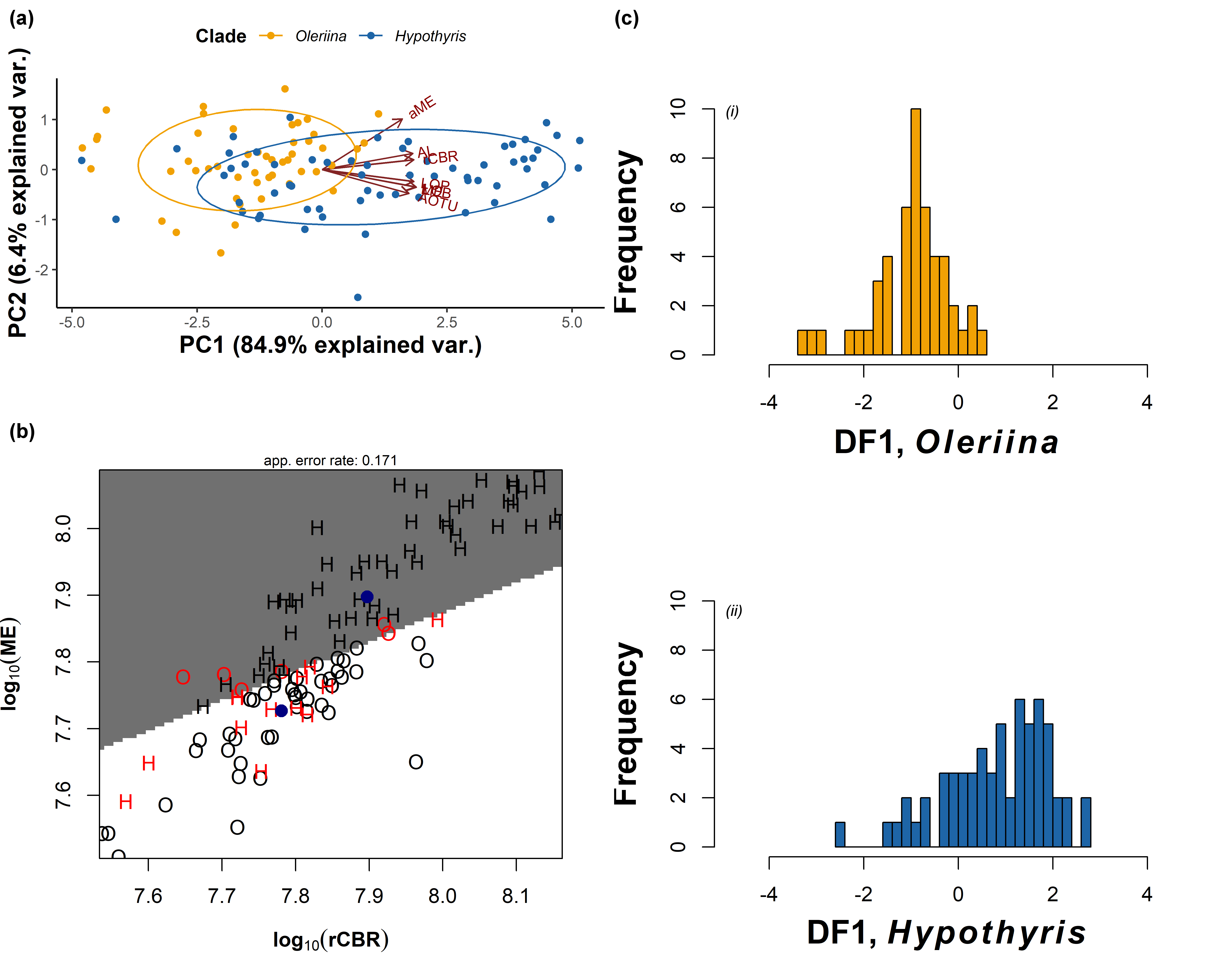

Supplement: Supplementary file 1 — Supplementary information [file EVO-76-1806-s002.tiff]

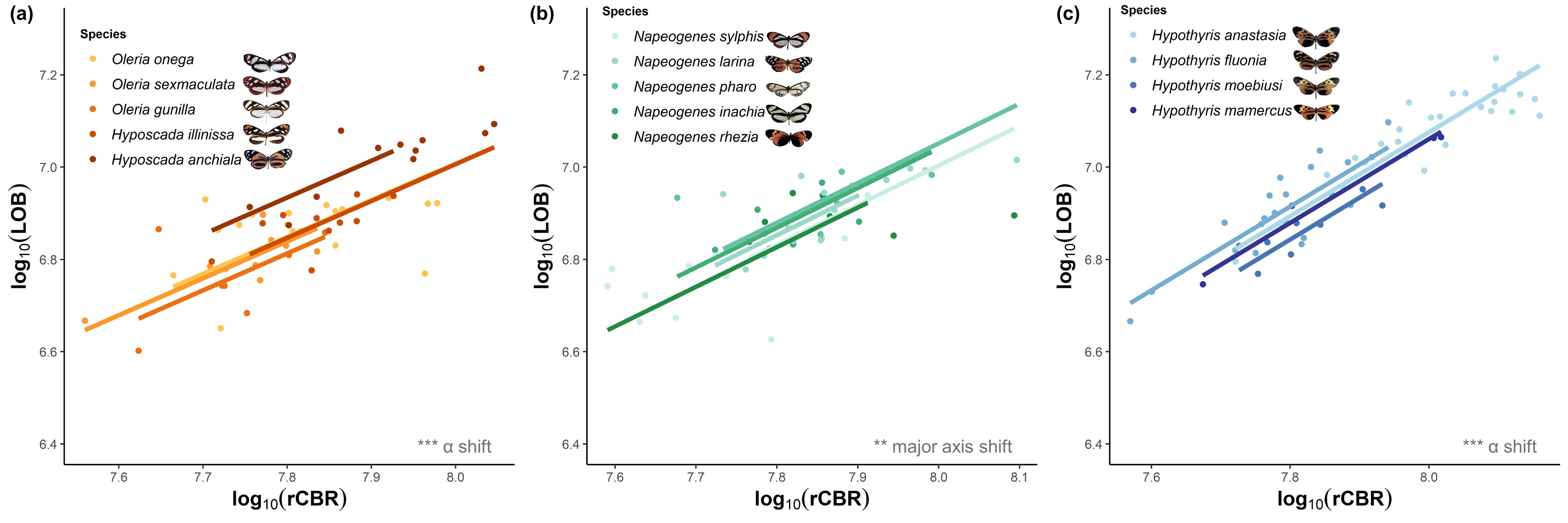

Supplement: Supplementary file 2 — Supplementary information [file EVO-76-1806-s001.tif]
