# Supplementary material for: Neuroanatomical shifts mirror patterns of ecological divergence in three diverse clades of mimetic butterflies
Source: Evolution. 2022 Jul 12;76(8):1806–20. doi: 10.1111/evo.14547 (PMC9540801; doi:10.1111/evo.14547)
Supplement: Supplementary file 3 — Figure S1: Multivariate analyses of brain morphology between monotypic clades (Hypothyris and Oleriina excluding Hyposcada anchiala). (a) Biplot of PC1 and PC2 from a principal component analyses for all neuropil (apart from the ventral lobula due to an abundance of zero values), in addition to rCBR to control for overall brain size. Vector lengths are proportional to the variance at that neuropil. (b) Partition plot denoting the classification of each individual to its respective clade based on a linear discriminant function analyses for medulla (ME) and rest of central brain (rCBR) total volume. Red signifies an incorrect classification. Means for each clade are plotted in blue. O = Oleriina (dark grey), H = Hypothyris (white). (c) Frequency histograms for the observations in each clade ((i) Oleriina, ii) Hypothyris) on the first linear discriminant function. Figure S2: Interspecific scaling relationships between the rest of central brain (rCBR) and the lobula (LOB) for Oleriina (a), Napeogenes (b), and Hypothyris (c). Results from the SMATR analysis are displayed where an ‘α shift’ denotes a grade‐shift in the relationship between the two variables, a ‘β shift’ denotes a shift in the allometric slope, and a ‘major axis shift’ signifies a main axis shift along a common slope. NS > 0.05, * P < 0.05, ** P < 0.01, *** P < 0.001 [file EVO-76-1806-s004.docx]

**Supplementary Figure Legends**

**Figure S1:** Multivariate analyses of brain morphology between monotypic clades (*Hypothyris* and Oleriina excluding *Hyposcada anchiala*). **(a)** Biplot of PC1 and PC2 from a principal component analyses for all neuropil (apart from the ventral lobula due to an abundance of zero values), in addition to rCBR to control for overall brain size. Vector lengths are proportional to the variance at that neuropil. **(b)** Partition plot denoting the classification of each individual to its respective clade based on a linear discriminant function analyses for medulla (ME) and rest of central brain (rCBR) total volume. Red signifies an incorrect classification. Means for each clade are plotted in blue. O = Oleriina (dark grey), H = *Hypothyris* (white). **(c)** Frequency histograms for the observations in each clade ((i) Oleriina, ii) *Hypothyris*) on the first linear discriminant function.

**Figure S2:** Interspecific scaling relationships between the rest of central brain (rCBR) and the lobula (LOB) for Oleriina **(a)**, *Napeogenes* **(b)**, and *Hypothyris* **(c).** Results from the SMATR analysis are displayed where an ‘α shift’ denotes a grade-shift in the relationship between the two variables, a ‘β shift’ denotes a shift in the allometric slope, and a ‘major axis shift’ signifies a main axis shift along a common slope. NS > 0.05, * P < 0.05, ** P < 0.01, *** P < 0.001
