# Supplementary material for: Neuroanatomical shifts mirror patterns of ecological divergence in three diverse clades of mimetic butterflies
Source: Evolution. 2022 Jul 12;76(8):1806–20. doi: 10.1111/evo.14547 (PMC9540801; doi:10.1111/evo.14547)
Supplement: Supplementary file 4 — Table S1: Raw Data Table S2: Linear Mixed Model Output Table S3: Standardised Major Axis Regression (SMATR) analysis Table S4: Principal Component Analyses Table S5: Discriminant Function Analyses Table S6: MCMCglmm Results [file EVO-76-1806-s003.pdf]

Table S1: Raw Data

Sub-tribe	Genus	Species	Mimicry	Flight height	Sex	Year	ID	log rCBR	log ME	log LOP	log LOB	log AL	log AOTU	log aME	log vLOB	log OL
Napeogenes	Napeogenes	rhezia	Coloured	0.300	Female	2011	35	7.766	7.731	6.532	6.838	6.730	5.918	4.572	4.345	7.808
Napeogenes	Napeogenes	rhezia	Coloured	0.300	Female	2012	87	7.944	7.760	6.578	6.852	6.964	5.968	4.974	4.285	7.836
Napeogenes	Napeogenes	rhezia	Coloured	0.300	Male	2012	98	8.093	7.897	6.694	6.896	7.201	6.141	NA	NA	7.963
Napeogenes	Napeogenes	inachia	Transparent	1.623	Male	2012	264	7.902	7.766	6.308	6.882	6.956	6.051	4.862	NA	7.833
Napeogenes	Napeogenes	inachia	Transparent	1.623	Male	2012	291	7.820	7.807	6.683	6.833	6.741	5.952	4.660	NA	7.880
Napeogenes	Napeogenes	larina	Coloured	1.325	Female	2011	25	7.893	7.848	6.679	6.960	6.895	6.032	4.909	4.280	7.926
Napeogenes	Napeogenes	larina	Coloured	1.325	Female	2011	30	7.855	7.700	6.522	6.846	6.806	5.856	4.848	3.916	7.782
Napeogenes	Napeogenes	larina	Coloured	1.325	Male	2011	128	7.983	7.963	6.750	6.993	7.070	6.217	5.131	4.260	8.031
Napeogenes	Napeogenes	larina	Coloured	1.325	Female	2011	198	7.762	7.732	6.548	6.778	6.868	5.714	4.876	4.073	7.803
Napeogenes	Napeogenes	larina	Coloured	1.325	Male	2011	224	8.097	7.906	6.538	7.015	7.006	6.147	4.847	NA	7.975
Napeogenes	Napeogenes	larina	Coloured	1.325	Male	2011	234	7.880	7.905	6.600	6.947	6.876	6.039	4.705	NA	7.969
Napeogenes	Napeogenes	larina	Coloured	1.325	Female	2012	97	7.935	7.918	6.702	6.972	6.972	6.037	5.105	4.054	7.988
Napeogenes	Napeogenes	larina	Coloured	1.325	Male	2012	153	7.965	7.927	6.719	6.997	7.056	6.088	5.206	3.880	7.999
Napeogenes	Napeogenes	larina	Coloured	1.325	Male	2012	196	7.871	7.822	6.580	6.907	6.852	6.072	4.891	0.000	7.894
Napeogenes	Napeogenes	larina	Coloured	1.325	Female	2012	239	7.820	7.755	6.564	6.840	6.860	5.938	4.839	4.130	7.830
Napeogenes	Napeogenes	larina	Coloured	1.325	Female	2012	257	7.734	7.844	6.625	6.941	6.969	6.047	5.052	4.190	7.919
Napeogenes	Napeogenes	larina	Coloured	1.325	Male	2012	268	7.814	7.758	6.607	6.868	6.714	5.910	4.776	NA	7.837
Napeogenes	Napeogenes	larina	Coloured	1.325	Female	2012	269	7.784	7.778	6.582	6.808	6.945	5.909	4.981	3.942	7.847
Napeogenes	Napeogenes	pharo	Transparent	1.264	Male	2011	206	7.791	7.784	6.536	6.824	6.755	5.928	4.705	NA	7.851
Napeogenes	Napeogenes	pharo	Transparent	1.264	Male	2012	65	7.677	7.879	6.697	6.934	6.988	6.027	5.078	NA	7.951
Napeogenes	Napeogenes	pharo	Transparent	1.264	Male	2012	67	7.880	7.914	6.719	6.990	6.946	6.038	4.855	NA	7.987
Napeogenes	Napeogenes	pharo	Transparent	1.264	Female	2012	142	7.854	7.790	6.526	6.855	6.912	5.972	4.959	4.352	7.859
Napeogenes	Napeogenes	pharo	Transparent	1.264	Female	2012	158	7.875	7.826	6.606	6.922	7.037	6.105	5.023	4.393	7.900
Napeogenes	Napeogenes	pharo	Transparent	1.264	Male	2012	231	7.991	7.890	6.706	6.984	6.976	6.141	5.156	3.756	7.966
Napeogenes	Napeogenes	sylphis	Transparent	1.081	Male	2012	182	7.793	7.640	6.338	6.627	6.791	5.774	4.797	NA	7.700
Napeogenes	Napeogenes	rhezia	Coloured	0.300	Male	2011	70	7.785	7.757	6.603	6.882	6.900	6.090	5.004	4.434	7.838
Napeogenes	Napeogenes	rhezia	Coloured	0.300	Male	2011	135	7.865	7.769	6.651	6.893	6.912	5.992	4.949	4.840	7.852
Napeogenes	Napeogenes	rhezia	Coloured	0.300	Male	2011	223	7.820	7.828	6.620	6.944	6.939	6.076	4.802	4.256	7.905
Napeogenes	Napeogenes	rhezia	Coloured	0.300	Female	2012	256	7.857	7.836	6.663	6.939	6.938	5.909	4.935	4.121	7.913
Napeogenes	Napeogenes	inachia	Transparent	1.623	Male	2011	22	7.724	7.702	6.568	6.821	6.805	6.049	4.671	NA	7.783
Napeogenes	Napeogenes	inachia	Transparent	1.623	Male	2011	85	7.856	7.882	6.640	6.966	6.951	6.110	4.874	NA	7.953
Napeogenes	Napeogenes	inachia	Transparent	1.623	Male	2011	240	7.776	7.829	6.624	6.908	6.936	6.121	4.921	NA	7.902
Napeogenes	Napeogenes	inachia	Transparent	1.623	Female	2012	313	7.784	7.815	6.531	6.856	6.930	5.912	4.769	3.910	7.880
Napeogenes	Napeogenes	sylphis	Transparent	1.081	Male	2011	92	7.837	7.747	6.559	6.862	6.903	6.052	4.912	NA	7.825
Napeogenes	Napeogenes	sylphis	Transparent	1.081	Male	2011	103	7.884	7.854	6.665	6.846	6.914	6.066	4.928	NA	7.920
Napeogenes	Napeogenes	sylphis	Transparent	1.081	Female	2012	49	7.638	7.579	6.345	6.722	6.668	5.776	4.696	3.996	7.658
Napeogenes	Napeogenes	sylphis	Transparent	1.081	Female	2012	107	7.738	7.678	6.551	6.764	6.806	5.868	4.811	4.284	7.757
Napeogenes	Napeogenes	sylphis	Transparent	1.081	Male	2012	139	7.596	7.603	6.453	6.779	6.656	5.777	4.817	NA	7.690
Napeogenes	Napeogenes	sylphis	Transparent	1.081	Female	2012	140	7.675	7.591	6.496	6.674	6.730	5.689	4.818	4.351	7.671
Napeogenes	Napeogenes	sylphis	Transparent	1.081	Male	2012	141	7.912	7.877	6.660	6.939	6.979	6.084	4.935	NA	7.948
Napeogenes	Napeogenes	sylphis	Transparent	1.081	Female	2012	157	7.591	7.507	6.456	6.742	6.629	5.816	4.818	4.237	7.608
Napeogenes	Napeogenes	sylphis	Transparent	1.081	Male	2012	195	7.631	7.594	6.444	6.665	6.699	5.810	4.775	NA	7.670
Napeogenes	Napeogenes	sylphis	Transparent	1.081	Male	2012	284	7.691	7.672	6.466	6.786	6.770	5.825	4.776	3.044	7.749
Napeogenes	Napeogenes	sylphis	Transparent	1.081	Male	2012	285	7.726	7.682	6.486	6.809	6.773	5.924	4.655	3.936	7.760
Oleriina	Oleria	gunilla	Transparent	0.425	Female	2011	108	7.647	7.778	6.533	6.866	6.761	5.936	4.701	4.426	7.850
Oleriina	Oleria	gunilla	Transparent	0.425	Male	2011	155	7.725	7.648	6.489	6.743	6.667	5.853	4.833	3.387	7.726
Oleriina	Oleria	gunilla	Transparent	0.425	Male	2012	45	7.752	7.626	6.476	6.684	6.706	6.066	4.736	3.687	7.700
Oleriina	Oleria	gunilla	Transparent	0.425	Female	2012	71	7.845	7.724	6.520	6.859	6.773	5.915	4.722	4.422	7.804
Oleriina	Oleria	gunilla	Transparent	0.425	Male	2012	109	7.802	7.733	6.479	6.810	6.739	5.956	4.814	NA	7.804
Oleriina	Oleria	gunilla	Transparent	0.425	Male	2012	335	7.623	7.586	6.320	6.602	6.650	5.716	4.763	4.424	7.651
Oleriina	Oleria	gunilla	Transparent	0.425	Female	2012	342	7.723	7.629	6.399	6.743	6.770	5.893	4.871	4.503	7.705
Oleriina	Oleria	onega	Transparent	0.610	Female	2012	21	7.978	7.802	6.636	6.922	6.897	6.038	4.903	4.683	7.882
Oleriina	Oleria	onega	Transparent	0.610	Female	2012	72	7.718	7.685	6.510	6.865	6.630	5.769	4.715	4.493	7.772
Oleriina	Oleria	onega	Transparent	0.610	Male	2012	179	7.967	7.828	6.408	6.921	6.924	6.187	4.745	NA	7.893
Oleriina	Oleria	onega	Transparent	0.610	Male	2012	296	7.857	7.806	6.442	6.830	6.776	6.029	4.767	NA	7.866
Oleriina	Oleria	sexmaculata	Transparent	0.567	Female	2011	21	7.781	7.786	6.518	6.842	6.792	5.969	4.830	4.826	7.854
Oleriina	Oleria	sexmaculata	Transparent	0.567	Male	2011	94	7.771	7.772	6.569	6.897	6.706	5.964	4.723	4.082	7.850
Oleriina	Oleria	sexmaculata	Transparent	0.567	Male	2011	95	7.708	7.668	6.444	6.785	6.627	5.876	4.756	3.898	7.744
Oleriina	Oleria	sexmaculata	Transparent	0.567	Male	2011	96	7.836	7.735	6.474	6.817	6.783	5.985	4.598	4.107	7.806
Oleriina	Oleria	sexmaculata	Transparent	0.567	Male	2011	109	7.762	7.687	6.436	6.787	6.702	5.895	4.435	3.739	7.760
Oleriina	Oleria	sexmaculata	Transparent	0.567	Male	2012	3	7.727	7.758	6.432	6.780	6.781	6.034	4.747	3.837	7.820
Oleriina	Oleria	sexmaculata	Transparent	0.567	Female	2012	12	7.560	7.508	6.296	6.667	6.601	5.836	4.729	NA	7.590
Oleriina	Oleria	sexmaculata	Transparent	0.567	Male	2012	22	7.799	7.751	6.458	6.830	6.809	6.014	4.824	3.682	7.820
Oleriina	Oleria	sexmaculata	Transparent	0.567	Female	2012	84	7.768	7.688	6.509	6.755	6.755	5.903	4.706	3.913	7.761
Oleriina	Oleria	assimilis	Transparent	0.300	Male	2012	311	7.546	7.543	6.368	6.629	6.512	5.765	4.695	3.989	7.619
Oleriina	Oleria	assimilis	Transparent	0.300	Male	2012	326	7.537	7.543	6.425	6.605	6.613	5.753	4.701	NA	7.620
Oleriina	Oleria	onega	Transparent	0.610	Male	2011	50	7.921	7.857	6.663	6.934	6.910	6.000	4.952	3.924	7.930
Oleriina	Oleria	onega	Transparent	0.610	Male	2011	61	7.816	7.726	6.635	6.860	6.833	5.963	4.992	4.048	7.812
Oleriina	Oleria	onega	Transparent	0.610	Female	2011	97	7.964	7.651	6.414	6.770	6.815	5.854	4.741	4.664	7.727
Oleriina	Oleria	onega	Transparent	0.610	Male	2011	98	7.721	7.553	6.347	6.651	6.533	5.725	4.547	3.765	7.628
Oleriina	Oleria	onega	Transparent	0.610	Male	2011	148	7.846	7.775	6.664	6.918	6.957	5.997	4.818	3.287	7.860
Oleriina	Oleria	onega	Transparent	0.610	Female	2011	149	7.807	7.756	6.560	6.862	6.923	5.941	4.848	4.464	7.832
Oleriina	Oleria	onega	Transparent	0.610	Female	2011	188	7.865	7.802	6.606	6.909	6.899	5.975	4.786	4.961	7.879
Oleriina	Oleria	onega	Transparent	0.610	Female	2011	189	7.816	7.745	6.546	6.862	6.909	5.941	4.926	4.424	7.822
Oleriina	Oleria	onega	Transparent	0.610	Male	2012	86	7.857	7.786	6.578	6.904	6.897	5.942	4.757	4.413	7.864
Oleriina	Oleria	onega	Transparent	0.610	Female	2012	138	7.743	7.743	6.519	6.875	6.765	6.179	4.849	4.395	7.821
Oleriina	Oleria	onega	Transparent	0.610	Female	2012	178	7.802	7.776	6.562	6.901	6.806	5.947	4.841	4.775	7.854
Oleriina	Oleria	onega	Transparent	0.610	Female	2012	196	7.800	7.747	6.561	6.875	6.898	5.938	4.775	4.265	7.827
Oleriina	Oleria	onega	Transparent	0.610	Male	2012	280	7.758	7.753	6.550	6.897	6.784	5.961	4.777	4.274	7.833
Oleriina	Oleria	onega	Transparent	0.610	Female	2012	281	7.665	7.667	6.433	6.765	6.705	5.821	4.659	4.196	7.741
Oleriina	Oleria	onega	Transparent	0.610	Female	2012	343	7.703	7.782	6.616	6.930	6.691	5.840	4.600	4.300	7.864
Oleriina	Hyposcada	anchiala	Coloured	1.126	Male	2011	31	7.835	7.846	6.545	6.936	6.829	5.985	4.536	3.836	7.915
Oleriina	Hyposcada	anchiala	Coloured	1.126	Male	2011	47	8.035	7.966	6.739	7.074	7.103	6.061	5.089	4.181	8.041
Oleriina	Hyposcada	anchiala	Coloured	1.126	Male	2011	105	7.756	7.760	6.575	6.913	6.727	5.960	4.747	4.377	7.843
Oleriina	Hyposcada	anchiala	Coloured	1.126	Female	2011	137	7.935	7.920	6.701	7.049	6.951	5.925	4.917	4.757	7.998
Oleriina	Hyposcada	anchiala	Coloured	1.126	Male	2012	76	7.864	7.917	6.666	7.079	6.874	5.993	4.790	3.755	7.997
Oleriina	Hyposcada	anchiala	Coloured	1.126	Male	2012	89	8.031	7.949	6.717	7.213	7.105	6.430	5.195	4.015	8.044
Oleriina	Hyposcada	anchiala	Coloured	1.126	Male	2012	119	7.908	7.923	6.693	7.042	6.923	6.072	4.871	3.467	7.999
Oleriina	Hyposcada	anchiala	Coloured	1.126	Female	2012	133	7.961	7.920	6.779	7.058	7.021	6.067	4.958	4.406	8.003
Oleriina	Hyposcada	anchiala	Coloured	1.126	Male	2012	200	7.950	7.934	6.680	7.018	6.926	6.061	4.901	4.019	8.005
Oleriina	Hyposcada	anchiala	Coloured	1.126	Female	2012	213	8.046	7.946	6.708	7.093	7.056	6.220	4.941	4.668	8.025
Oleriina	Hyposcada	anchiala	Coloured	1.126	Male	2012	214	7.802	7.782	6.615	6.874	6.814	5.899	4.794	3.863	7.859
Oleriina	Hyposcada	anchiala	Coloured	1.126	Female	2012	235	7.952	7.917	6.660	7.036	6.907	5.984	4.757	4.704	7.992
Oleriina	Hyposcada	illinissa	Transparent	0.600	Male	2011	19	7.771	7.766	6.504	6.879	6.622	6.027	4.824	4.279	7.840
Oleriina	Hyposcada	illinissa	Transparent	0.600	Male	2011	218	7.883	7.786	6.636	6.883	6.737	6.039	4.958	4.079	7.864
Oleriina	Hyposcada	illinissa	Transparent	0.600	Female	2012	10	7.710	7.692	6.533	6.796	6.636	5.697	4.931	4.363	7.771
Oleriina	Hyposcada	illinissa	Transparent	0.600	Female	2012	43	7.863	7.778	6.587	6.880	6.914	5.924	4.858	4.728	7.854
Oleriina	Hyposcada	illinissa	Transparent	0.600	Female	2012	44	7.883	7.821	6.612	6.941	6.905	5.792	4.867	4.739	7.899
Oleriina	Hyposcada	illinissa	Transparent	0.600	Female	2012	85	7.795	7.759	6.589	6.896	6.766	5.777	4.800	4.402	7.840
Oleriina	Hyposcada	illinissa	Transparent	0.600	Female	2012	95	7.829	7.797	6.543	6.776	6.971	5.780	4.950	4.769	7.859
Oleriina	Hyposcada	illinissa	Transparent	0.600	Female	2012	131	7.835	7.771	6.564	6.890	6.791	5.958	4.862	4.474	7.849
Oleriina	Hyposcada	kena	Transparent	0.500	Female	2012	23	7.670	7.684	6.437	6.788	6.628	5.885	4.522	4.653	7.758
Oleriina	Hyposcada	illinissa	Transparent	0.600	Female	2012	117	7.849	7.765	6.562	6.862	6.827	5.895	4.925	4.498	7.840
Oleriina	Hyposcada	illinissa	Transparent	0.600	Female	2012	180	7.926	7.843	6.670	6.938	6.933	5.997	5.063	4.732	7.920
Oleriina	Hyposcada	kena	Transparent	0.500	Male	2012	297	7.738	7.744	6.589	6.874	6.658	5.944	4.493	3.386	7.825
Hypothyris	Hypothyris	anastasia	Coloured	2.364	Female	2011	51	7.931	7.936	6.668	7.051	7.015	6.136	4.989	3.433	8.010
Hypothyris	Hypothyris	anastasia	Coloured	2.364	Male	2011	64	8.016	8.033	6.794	7.110	7.113	6.210	4.969	3.556	8.104
Hypothyris	Hypothyris	anastasia	Coloured	2.364	Male	2011	65	7.957	8.011	6.858	7.082	6.985	6.126	5.000	0.000	8.086
Hypothyris	Hypothyris	anastasia	Coloured	2.364	Female	2011	80	7.853	7.861	6.675	6.983	6.971	6.010	5.000	4.394	7.940
Hypothyris	Hypothyris	anastasia	Coloured	2.364	Female	2011	81	7.894	7.950	6.712	7.020	7.035	6.063	4.900	4.121	8.021
Hypothyris	Hypothyris	anastasia	Coloured	2.364	Male	2011	89	7.785	7.780	6.723	6.878	6.848	6.078	4.641	0.000	7.864
Hypothyris	Hypothyris	anastasia	Coloured	2.364	Male	2011	113	8.034	8.041	6.821	7.160	7.122	6.155	5.160	4.331	8.118
Hypothyris	Hypothyris	anastasia	Coloured	2.364	Female	2011	115	7.955	7.966	6.767	7.055	7.106	6.109	4.952	4.556	8.041
Hypothyris	Hypothyris	anastasia	Coloured	2.364	Male	2011	161	8.131	8.085	6.855	7.202	7.192	6.310	5.118	4.099	8.161
Hypothyris	Hypothyris	anastasia	Coloured	2.364	Male	2011	182	8.090	8.041	6.832	7.122	7.107	6.197	5.079	0.000	8.114
Hypothyris	Hypothyris	anastasia	Coloured	2.364	Male	2011	183	7.993	7.864	6.665	6.993	7.012	6.080	4.976	4.314	7.943
Hypothyris	Hypothyris	anastasia	Coloured	2.364	Male	2011	227	8.075	8.004	6.822	7.130	7.062	6.218	5.081	0.000	8.083
Hypothyris	Hypothyris	anastasia	Coloured	2.364	Male	2012	39	8.003	8.011	6.858	7.108	6.985	6.126	5.000	4.808	8.089
Hypothyris	Hypothyris	anastasia	Coloured	2.364	Female	2012	60	8.120	8.004	6.779	7.120	7.198	6.120	5.035	4.563	8.080
Hypothyris	Hypothyris	anastasia	Coloured	2.364	Male	2012	78	8.053	8.073	6.815	7.160	7.021	6.248	5.124	0.000	8.144
Hypothyris	Hypothyris	anastasia	Coloured	2.364	Male	2012	102	8.094	8.070	6.834	7.141	7.134	6.259	5.221	3.662	8.141
Hypothyris	Hypothyris	anastasia	Coloured	2.364	Male	2012	103	8.131	8.063	6.882	7.158	7.202	6.290	5.202	4.143	8.139
Hypothyris	Hypothyris	anastasia	Coloured	2.364	Male	2012	126	7.875	7.866	6.601	6.958	6.924	6.111	4.876	3.807	7.937
Hypothyris	Hypothyris	anastasia	Coloured	2.364	Male	2012	132	8.095	8.036	6.842	7.175	7.144	6.300	5.031	3.582	8.116
Hypothyris	Hypothyris	anastasia	Coloured	2.364	Female	2012	135	7.907	7.884	6.684	7.010	6.903	6.021	4.815	4.333	7.963
Hypothyris	Hypothyris	anastasia	Coloured	2.364	Male	2012	162	8.107	8.055	6.805	7.169	7.070	6.214	5.117	0.000	8.130
Hypothyris	Hypothyris	anastasia	Coloured	2.364	Female	2012	163	8.153	8.010	6.862	7.148	7.140	6.173	5.086	4.554	8.092
Hypothyris	Hypothyris	anastasia	Coloured	2.364	Female	2012	189	8.159	8.020	6.846	7.111	7.150	6.188	5.190	4.730	8.097
Hypothyris	Hypothyris	anastasia	Coloured	2.364	Male	2012	199	8.097	8.064	6.865	7.236	7.084	6.335	4.965	4.224	8.147
Hypothyris	Hypothyris	anastasia	Coloured	2.364	Female	2012	240	8.024	7.970	6.758	7.048	7.052	6.210	4.962	4.524	8.043
Hypothyris	Hypothyris	anastasia	Coloured	2.364	Female	2012	259	7.971	8.056	6.861	7.140	6.886	6.261	5.048	4.101	8.131
Hypothyris	Hypothyris	fluonia	Coloured	1.308	Female	2011	15	7.778	7.794	6.612	6.901	6.818	5.978	4.768	4.347	7.871
Hypothyris	Hypothyris	fluonia	Coloured	1.308	Male	2011	75	7.941	8.066	6.800	7.097	7.047	6.213	5.186	NA	8.131
Hypothyris	Hypothyris	fluonia	Coloured	1.308	Female	2011	76	7.569	7.591	6.400	6.666	6.552	5.637	4.602	3.873	7.664
Hypothyris	Hypothyris	fluonia	Coloured	1.308	Female	2011	129	7.830	7.910	6.744	7.000	6.849	6.009	4.824	3.922	7.986
Hypothyris	Hypothyris	fluonia	Coloured	1.308	Male	2011	136	7.786	7.893	6.673	6.977	6.743	6.021	4.771	NA	7.966
Hypothyris	Hypothyris	anastasia	Coloured	2.364	Female	2011	138	7.965	7.950	6.715	7.027	7.028	6.152	4.930	4.392	8.021
Hypothyris	Hypothyris	fluonia	Coloured	1.308	Female	2011	164	7.601	7.649	6.436	6.731	6.556	5.795	4.485	4.000	7.722
Hypothyris	Hypothyris	fluonia	Coloured	1.308	Male	2011	197	7.843	7.947	6.715	7.036	6.871	6.127	4.922	4.281	8.020
Hypothyris	Hypothyris	fluonia	Coloured	1.308	Male	2011	207	7.917	7.951	6.755	7.022	6.921	6.168	4.904	NA	8.023
Hypothyris	Hypothyris	fluonia	Coloured	1.308	Female	2011	208	7.819	7.793	6.575	6.847	6.848	5.876	4.756	4.189	7.863
Hypothyris	Hypothyris	fluonia	Coloured	1.308	Female	2011	233	7.816	7.721	6.556	6.833	6.816	5.868	4.776	4.081	7.800
Hypothyris	Hypothyris	fluonia	Coloured	1.308	Male	2012	73	7.794	7.884	6.573	6.941	6.776	6.036	4.752	NA	7.949
Hypothyris	Hypothyris	fluonia	Coloured	1.308	Female	2012	91	7.763	7.814	6.544	6.889	6.806	5.941	4.605	4.342	7.883
Hypothyris	Hypothyris	fluonia	Coloured	1.308	Female	2012	122	7.750	7.780	6.566	6.814	6.752	5.918	4.644	4.344	7.848
Hypothyris	Hypothyris	fluonia	Coloured	1.308	Male	2012	187	7.770	7.890	6.618	6.938	6.822	6.051	4.766	3.910	7.957
Hypothyris	Hypothyris	fluonia	Coloured	1.308	Male	2012	233	7.705	7.767	6.571	6.880	6.658	5.974	4.698	4.126	7.844
Hypothyris	Hypothyris	fluonia	Coloured	1.308	Male	2012	238	7.758	7.797	6.598	6.876	6.703	5.975	4.673	3.998	7.870
Hypothyris	Hypothyris	fluonia	Coloured	1.308	Female	2012	255	7.721	7.747	6.532	6.820	6.742	5.847	4.712	4.363	7.819
Hypothyris	Hypothyris	fluonia	Coloured	1.308	Male	2012	266	7.794	7.844	6.606	6.881	6.814	5.996	4.898	4.219	7.911
Hypothyris	Hypothyris	fluonia	Coloured	1.308	Female	2012	302	7.887	7.894	6.618	6.918	6.925	6.004	4.879	4.441	7.958
Hypothyris	Hypothyris	fluonia	Coloured	1.308	Male	2012	303	7.883	7.933	6.672	7.011	6.893	6.083	4.853	4.562	8.004
Hypothyris	Hypothyris	fluonia	Coloured	1.308	Female	2012	338	7.860	7.831	6.609	6.941	6.793	6.004	4.902	3.800	7.907
Hypothyris	Hypothyris	moebiusi	Coloured	1.750	Male	2011	114	7.933	7.871	6.715	6.917	7.009	6.131	4.975	4.562	7.944
Hypothyris	Hypothyris	anastasia	Coloured	2.364	Male	2011	225	7.721	7.748	6.538	6.795	6.729	5.967	4.817	3.874	7.818
Hypothyris	Hypothyris	moebiusi	Coloured	1.750	Female	2012	100	7.767	7.729	6.442	6.837	6.783	5.905	4.722	4.008	7.801
Hypothyris	Hypothyris	moebiusi	Coloured	1.750	Male	2012	216	7.843	7.763	6.519	6.876	6.836	5.992	4.677	4.119	7.838
Hypothyris	Hypothyris	moebiusi	Coloured	1.750	Male	2012	218	7.904	7.865	6.656	6.952	6.766	6.133	4.985	3.323	7.939
Hypothyris	Hypothyris	moebiusi	Coloured	1.750	Male	2012	270	7.801	7.731	6.550	6.811	6.835	5.995	4.978	NA	7.806
Hypothyris	Hypothyris	moebiusi	Coloured	1.750	Male	2012	305	7.809	7.778	6.602	6.879	6.754	6.025	4.793	NA	7.855
Hypothyris	Hypothyris	moebiusi	Coloured	1.750	Male	2012	317	7.726	7.701	6.495	6.829	6.751	5.871	4.858	NA	7.780
Hypothyris	Hypothyris	moebiusi	Coloured	1.750	Female	2012	318	7.753	7.636	6.350	6.769	6.757	5.824	4.684	3.618	7.711
Hypothyris	Hypothyris	mamercus	Coloured	2.642	Female	2011	184	8.017	7.990	6.748	7.065	7.075	6.238	5.035	NA	8.061
Hypothyris	Hypothyris	mamercus	Coloured	2.642	Male	2011	6	8.007	8.004	6.766	7.063	7.014	6.259	4.876	4.876	8.073
Hypothyris	Hypothyris	mamercus	Coloured	2.642	Female	2011	221	7.802	7.892	6.690	6.916	6.942	6.253	4.727	NA	7.960
Hypothyris	Hypothyris	mamercus	Coloured	2.642	Female	2011	238	7.674	7.734	6.571	6.746	6.831	5.864	4.771	3.913	7.803
Napeogenes	Napeogenes	larina	Coloured	1.325	Female	2011	209	7.830	7.923	6.669	6.981	6.951	6.127	4.521	NA	7.991
Napeogenes	Napeogenes	larina	Coloured	1.325	Male	2012	56	7.854	7.858	6.664	6.842	7.007	6.068	4.886	NA	7.923
Napeogenes	Napeogenes	larina	Coloured	1.325	Male	2012	58	7.815	7.858	6.664	6.842	7.007	6.068	4.886	NA	7.923
Napeogenes	Napeogenes	larina	Coloured	1.325	Female	2012	59	7.858	7.865	6.583	6.944	6.994	5.975	4.787	NA	7.934

UOB Open

Table S2: Linear Mixed Model Output

Key
P < 0.05
P < 0.01
P < 0.001

A) Interclade comparisons (Neuropil ~ rCBR + Clade + (1 | Species) + (1 | Sex))

Monotypic clades (with *H. anchiala*) *(Statistical power for Clade = 73.9%, powerSim function, nsim = 1000)*

Neuropil	*LogLh - without clade*	*LogLh - with clade*	*ChiSq*	*P*
ME	177.420	180.520	6.204	0.013
LOP	154.580	157.220	5.284	0.022
LOB	169.600	170.730	2.262	0.133
aME	101.710	101.810	0.203	0.652
vLOB	-34.412	-32.255	4.315	0.038
AL	162.790	165.170	4.752	0.029
AOTU	128.360	130.980	5.255	0.022
OL	179.890	182.780	5.780	0.016

Monotypic clades (without *H. anchiala*) *(Statistical power for Clade= 88.00%, powerSim function, nsim = 1000)*

Neuropil	*LogLh - without clade*	*LogLh - with clade*	*ChiSq*	*P*
ME	156.160	160.560	8.787	0.003
LOP	136.100	139.500	6.801	0.009
LOB	153.030	156.530	7.003	0.008
aME	93.653	93.676	0.046	0.831
vLOB	-32.161	-29.840	4.641	0.031
AL	143.310	146.170	5.731	0.017
AOTU	119.470	121.890	4.833	0.028
OL	158.380	162.710	7.614	0.003

All three clades (with *H. anchiala*) *(Statistical power for Clade = 64.7%, powerSim function, nsim = 1000)*

Neuropil	*LogLh - without clade*	*LogLh - with clade*	*ChiSq*	*P*
ME	241.480	244.810	6.669	0.036
LOP	195.960	199.560	7.202	0.027
LOB	227.320	229.220	3.804	0.149
aME	126.240	128.670	4.852	0.088
vLOB	-292.410	-285.690	13.456	0.001
AL	206.150	215.230	18.150	<0.001
AOTU	177.230	180.630	6.804	0.033
OL	244.580	247.820	6.489	0.039

All three clades (without *H. anchiala*) *(Statistical power for Clade = 84 %, powerSim function, nsim = 1000)*

Neuropil	*LogLh - without clade*	*LogLh - with clade*	*ChiSq*	*P*
ME	204.790	209.320	9.059	0.011
LOP	164.780	169.500	9.431	0.009
LOB	200.190	204.480	8.585	0.014
aME	113.490	115.850	4.722	0.094
vLOB	-39.352	-36.832	5.040	0.080
AL	178.030	185.310	14.554	0.001
AOTU	157.850	160.730	5.753	0.056
OL	207.750	212.360	9.213	0.010

Hypothyris* versus *H. anchiala

Neuropil	*LogLh - without clade*	*LogLh - with clade*	*ChiSq*	*P*
ME	114.890	114.940	0.088	0.766
LOP	106.800	106.890	0.189	0.664
LOB	116.280	116.820	1.082	0.298
aME	69.891	71.020	2.256	0.133
vLOB	-20.709	-20.684	0.049	0.825
AL	109.650	109.800	0.299	0.584
AOTU	84.081	84.780	1.400	0.237
OL	117.690	117.710	0.036	0.849

Bi) Interspecific comparisons (Neuropil ~ rCBR + Species + (1 | Sex))

***Oleriina* (with *H. anchiala*) (Statistical power = 98.4%)**

Neuropil	*LogLh-without species*	*LogLh - with species*	*Chisq*	*P*
ME	87.398	99.537	24.279	<0.001
LOP	71.958	81.271	18.625	0.005
LOB	73.219	88.159	29.881	<0.001
aME	44.454	54.017	19.125	0.004
vLOB	-65.950	-60.090	11.720	0.069
AL	76.820	79.427	5.203	0.518
AOTU	56.568	60.761	8.386	0.211
OL	87.176	100.260	26.167	<0.001

***Napeogenes* (Statistical power = 96.10%)**

Neuropil	*Loglh - without species*	*Loglh - with species*	*Chisq*	*P*
ME	60.838	69.871	18.066	0.001
LOP	49.416	53.462	8.091	0.088
LOB	62.222	68.968	13.491	0.009
aME	28.677	30.761	4.166	0.384
vLOB	-80.527	-75.757	11.022	0.026
AL	54.445	56.469	4.047	0.400
AOTU	48.968	52.632	7.328	0.120
OL	62.409	71.390	17.961	0.001

***Hypothyris* (Statistical power = 99.28%)**

Neuropil	*LogLh- without species*	*LogLh - with species*	*ChiSq*	*P*
ME	88.745	101.181	24.871	<0.001
LOP	82.228	92.325	20.194	<0.001
LOB	96.247	104.479	16.462	0.001
aME	65.003	65.426	0.847	0.838
vLOB	-118.290	-116.360	3.868	0.276
AL	88.010	94.514	13.006	0.005
AOTU	74.360	81.844	14.968	0.002
OL	90.805	103.265	24.921	<0.001

Bii) Interspecific comparisons within Oleriina (minus one species)

***Oleriina* (without *H. anchiala*) (Statistical power = 41%...)**

Neuropil	*LogLh-without species*	*LogLh-with species*	*ChiSq*	*P*
ME	73.687	76.510	5.646	0.342
LOP	57.997	61.981	7.969	0.158
LOB	65.251	70.824	11.147	0.049
aME	36.725	48.547	23.644	<0.001
vLOB	-54.780	-51.241	7.079	0.215
AL	59.360	60.665	2.610	0.760
AOTU	47.527	51.236	7.419	0.191
OL	73.931	77.145	6.429	0.267

***Oleriina* (without *H. illinissa*)**

Neuropil	*LogLh-without species*	*LogLh-with species*	*ChiSq*	*P*
ME	69.030	79.213	20.366	0.001
LOP	56.430	64.346	15.832	0.007
LOB	58.443	71.198	25.510	<0.001
aME	37.112	42.447	10.671	0.058
vLOB	-58.540	-53.858	9.364	0.095
AL	67.051	69.353	4.604	0.466
AOTU	50.840	51.987	2.296	0.807
OL	68.787	79.728	21.882	0.001

***Oleriina* (without *O. gunilla*)**

Neuropil	*LogLh-without sp*	*LogLh-with sp*	*ChiSq*	*P*
ME	80.165	91.402	22.474	<0.001
LOP	63.454	71.549	16.189	0.006
LOB	67.122	81.223	28.201	<0.001
aME	37.705	46.207	17.003	0.004
vLOB	-60.004	-55.102	9.805	0.081
AL	67.700	69.656	3.911	0.562
AOTU	49.678	53.486	7.617	0.179
OL	79.925	92.057	24.264	<0.001

***Oleriina* (without *O. sexmaculata*)**

Neuropil	*LogLh-witho*	*LogLh-with s*	*ChiSq*	*P*
ME	72.651	85.713	26.125	<0.001
LOP	60.415	67.691	14.553	0.012
LOB	59.821	72.836	26.030	<0.001
aME	40.238	49.637	18.797	0.002
vLOB	-44.531	-36.090	16.882	0.005
AL	63.193	65.114	3.842	0.572
AOTU	45.887	48.924	6.073	0.299
OL	72.215	85.741	27.052	<0.001

***Oleriina* (without *H. kena*)**

Neuropil	*LogLh-without species*	*LogLh-with species*	*ChiSq*	*P*
ME	83.890	95.354	22.928	<0.001
LOP	69.384	78.538	18.308	0.003
LOB	70.392	84.477	28.169	<0.001
aME	45.975	51.386	10.822	0.055
vLOB	-63.963	-57.888	12.149	0.033
AL	74.035	75.829	3.589	0.610
AOTU	53.974	57.784	7.621	0.178
OL	83.727	96.102	24.750	<0.001

***Oleriina* (without *O. assimilis*)**

Neuropil	*LogLh-without species*	*LogLh-with species*	*ChiSq*	*P*
ME	83.630	95.293	23.326	<0.001
LOP	69.007	77.843	17.672	0.003
LOB	69.988	84.313	28.651	<0.001
aME	42.664	51.265	17.203	0.004
vLOB	-53.342	-49.148	8.388	0.136
AL	74.252	76.521	4.537	0.475
AOTU	53.745	57.784	8.078	0.152
OL	83.399	95.992	25.186	<0.001

***Oleriina* (without *O. onega*)**

Neuropil	*LogLh-without sp*	*LogLh-with sp*	*ChiSq*	*P*
ME	68.137	77.000	17.727	0.003
LOP	62.038	72.639	21.202	0.001
LOB	55.601	66.795	22.388	<0.001
aME	28.387	36.910	17.047	0.004
vLOB	-51.833	-46.935	9.797	0.081
AL	57.807	61.327	7.040	0.218
AOTU	40.091	44.349	8.515	0.130
OL	68.411	78.536	20.250	0.001

Ci) Test for Sex:Species interactions (Neuropil ~ rCBR + Sex*Species)

Neuropil	*RSS-without interaction*	*RSS - with interactiion*	*F*	*P*
ME	0.432	0.381	1.296	0.217
LOP	0.776	0.694	1.169	0.306
LOB	0.529	0.464	1.371	0.175
aME	1.833	1.706	0.728	0.744
vLOB	287.790	231.310	2.197	0.011
AL	0.692	0.625	1.053	0.406
AOTU	0.982	0.893	0.981	0.476
OL	0.416	0.367	1.324	0.201

Cii) Sexual dimorphism comparisons (Neuropil ~ rCBR + Sex + (1 | Species))

Neuropil	*LogLh-without sex*	*LogLh - with sex*	*Chisq*	*P*
ME	240.440	243.020	5.146	0.023
LOP	195.960	196.650	1.392	0.238
LOB	227.320	227.650	0.650	0.420
aME	126.240	126.310	0.142	0.706
vLOB	-302.540	-289.300	26.497	<0.001
AL	205.590	207.600	4.035	0.045
AOTU	169.570	180.230	21.316	<0.001
OL	243.740	246.040	4.615	0.032

D) Mimicry (Tiger-stripe vs. Transparent) comparisons (Neuropil ~ rCBR + Mimicry + (1|Species) + (1 | Sex))

Neuropil	*LogLh-without mimicry*	*LogLh - with mimicry*	*Chisq*	*P*
ME	241.480	244.840	6.721	0.010
LOP	195.960	200.110	8.304	0.004
LOB	227.320	230.520	6.405	0.011
aME	126.240	126.250	0.021	0.886
vLOB	-292.410	-292.340	0.147	0.702
AL	206.150	207.120	1.923	0.166
AOTU	177.230	178.590	2.723	0.099
OL	244.580	248.100	7.038	0.008

E) Covariance analysis between optic lobe neuropil

i) t-values

Neuropil	*ME*	*LOP*	*LOB*	*aME*	*AL*	*AOTU*
ME		5.534	8.011	2.272	4.238	2.843
LOP	5.838		3.220	3.525	0.071	-1.168
LOB	8.004	2.916		0.376	-1.688	2.783
aME	-2.322	3.383	0.241		5.374	0.948
AL	4.215	-0.208	-1.721	5.348		0.433
AOTU	2.843	-1.031	2.756	1.079	0.376	

i) p values

Neuropil	*ME*	*LOP*	*LOB*	*aME*	*AL*	*AOTU*
ME		<0.001	<0.001	0.024	<0.001	0.005
LOP	<0.001		0.002	<0.001		
LOB	<0.001	0.004		0.707	0.093	0.006
aME	0.022	0.001	0.810		<0.001	0.345
AL	<0.001	0.836	0.087	<0.001		0.665
AOTU	0.005	0.304	0.007	0.282	0.708	

iii) associations with rCBR and Species

	**rCBR**		***Species***	
Neuropil	*t-value*	*p-value*	*F*	*p-value*
ME	1.855	0.065	3.499	<0.001
LOP	-2.014	0.046	1.176	0.296
LOB	2.759	0.006	3.216	<0.001
aME	1.300	0.195	2.193	0.009
AL	5.387	<0.001	2.657	0.001
AOTU	1.096	0.275	2.749	0.001

UOB Open

Table S3: Standardised Major Axis Regression (SMATR) analysis

Key

P < 0.05
P < 0.01
P < 0.001

A) i) Monotypic clade comparisons (without *H. anchiala*)

Neuropil	**Slope shift** (Neuropil ~ rCBR*Clade) Likelihood ratio	p	**Elevation shift** (Neuropil ~rCBR + Clade, type = "elevation") Wald statistic	p	**Major axis shift** (Neuropil ~ rCBR + Clade, type = "shift") Wald statistic	p
ME	0.394	0.530	36.180	<0.001		
LOP	1.335	0.248	18.470	<0.001		
LOB	0.324	0.569	12.420	<0.001		
vLOB	12.230	<0.001				
AL	0.447	0.504	1.296	0.255	24.620	<0.001
AOTU	0.078	0.780	5.189	0.023		
OL	0.543	0.461	35.760	<0.001		

A) ii) Monotypic clade comparisons (with *H. anchiala*)

Neuropil	**Slope shift** (Neuropil ~ rCBR*Clade) Likelihood ratio	p	**Elevation shift** (Neuropil ~rCBR + Clade, type = "elevation") Wald statistic	p	**Major axis shift** (Neuropil ~ rCBR + Clade, type = "shift") Wald statistic	p
ME	0.430	0.512	25.530	<0.001		
LOP	1.673	0.196	17.640	<0.001		
vLOB	8.467	0.004				
AL	0.486	0.486	1.503	0.220	15.210	<0.001
AOTU	0.070	0.791	8.885	0.003		
OL	0.564	0.453	24.060	<0.001		

B) Within-clade comparisons

Oleriina

Neuropil	**Slope shift** (Neuropil ~ rCBR*Species) Likelihood ratio	p	**Elevation shift** (Neuropil ~rCBR + Species, type = "elevation") Wald statistic	p	**Major axis shift** (Neuropil ~ rCBR + Species, type = "shift") Wald statistic	p
ME	3.205	0.524	35.090	<0.001		
LOP	3.690	0.450	7.631	0.106	52.330	<0.001
LOB	4.139	0.388	19.560	<0.001		
aME	3.143	0.534	11.770	0.019		
vLOB	4.077	0.396	21.670	<0.001		
OL	3.444	0.487	41.320	<0.001		

Napeogenes

Neuropil	**Slope shift** (Neuropil ~ rCBR*Species) Likelihood ratio	p	**Elevation shift** (Neuropil ~rCBR + Species, type = "elevation") Wald statistic	p	**Major axis shift** (Neuropil ~ rCBR + Species, type = "shift") Wald statistic	p
ME	3.745	0.442	7.668	0.105	15.810	0.003
LOB	4.552	0.336	1.197	0.879	17.750	0.001
vLOB	11.100	0.025				
OL	3.403	0.493	6.835	0.145	15.930	0.003

Hypothyris

Neuropil	**Slope shift** (Neuropil ~ rCBR*Species) Likelihood ratio	p	**Elevation shift** (Neuropil ~rCBR + Species, type = "elevation") Wald statistic	p	**Major axis shift** (Neuropil ~ rCBR + Species, type = "shift") Wald statistic	p
ME	7.367	0.061	35.060	<0.001		
LOP	11.230	0.011				
LOB	3.835	0.280	13.530	0.004		
AL	6.802	0.078	2.392	0.495	69.810	<0.001
AOTU	8.933	0.030				
OL	7.217	0.065	56.270	<0.001		

C) Pairwise interspecifc comparisons

i) *Oleriina*

Test statistics

Elevation shift (Neuropil ~ rCBR + Species, type = "elevation") — *Pairwise comparisons*

Neuropil	onega.sexmaculata	onega.gunilla	onega.anchiala	onega.illinissa	sexmaculata.gunila	sexmaculata.anchiala	sexmaculata.illinissa	gunilla.anchiala	gunilla.illinissa	anchiala.illinissa
ME	0.041	3.385	24.147	0.750	1.951	5.866	0.021	16.408	5.330	27.336
LOB	3.023	4.105	10.651	0.090	1.163	6.653	0.583	5.544	1.124	9.844
aME	0.033	2.079	1.210	4.525	0.139	1.771	0.561	3.749	0.021	8.533
vLOB	1.582	0.282	10.111	0.589	1.949	4.816	3.055	5.734	0.044	16.611
OL	0.061	5.491	24.774	0.634	2.331	6.596	0.253	17.087	7.368	28.967

Major axis shift (Neuropil ~ rCBR + Species, type = "shift")

Neuropil	onega.sexmaculata	onega.gunilla	onega.anchiala	onega.illinissa	sexmaculata.gunila	sexmaculata.anchiala	sexmaculata.illinissa	gunilla.anchiala	gunilla.illinissa	anchiala.illinissa
LOP	7.899	6.160	16.958	1.318	0.184	42.366	15.630	30.537	11.231	9.910

P values (adjusted)

Elevation shift (Neuropil ~ rCBR + Species, type = "elevation") — *Pairwise comparisons*

Neuropil	onega.sexmaculata	onega.gunilla	onega.anchiala	onega.illinissa	sexmaculata.gunilla	sexmaculata.anchiala	sexmaculata.illinissa	gunilla.anchiala	gunilla.illinissa	anchiala.illinissa
ME	1.000	0.494	<0.001	0.992	0.830	0.144	1.000	<0.001	0.191	<0.001
LOB	0.575	0.354	0.011	1.000	0.963	0.095	0.997	0.171	0.967	0.017
aME	1.000	0.802	0.958	0.288	1.000	0.868	0.998	0.419	1.000	0.034
vLOB	0.903	1.000	0.015	0.997	0.831	0.249	0.568	0.155	1.000	<0.001
OL	1.000	0.176	<0.001	0.996	0.742	0.098	1.000	<0.001	0.064	<0.001

Major axis shift (Neuropil ~ rCBR + Species, type = "shift")

Neuropil	onega.sexmaculata	onega.gunilla	onega.anchiala	onega.illinissa	sexmaculata.gunilla	sexmaculata.anchiala	sexmaculata.illinissa	gunilla.anchiala	gunilla.illinissa	anchiala.illinissa
LOP	0.048	0.123	<0.001	0.944	1.000	<0.001	0.001	<0.001	0.008	0.016

***P values* (non-adjusted)**

Elevation shift (Neuropil ~ rCBR + Species, type = "elevation") — *Pairwise comparisons*

Neuropil	onega.sexmaculata	onega.gunilla	onega.anchiala	onega.illinissa	sexmaculata.gunilla	sexmaculata.anchiala	sexmaculata.illinissa	gunilla.anchiala	gunilla.illinissa	anchiala.illinissa
ME	0.840	0.066	<0.001	0.386	0.162	0.015	0.885	<0.001	0.021	<0.001
LOB	0.082	0.043	0.001	0.764	0.281	0.010	0.445	0.019	0.289	0.002
aME	0.856	0.149	0.271	0.033	0.709	0.183	0.454	0.053	0.886	0.003
vLOB	0.208	0.596	0.001	0.443	0.163	0.028	0.080	0.017	0.834	<0.001
OL	0.804	0.019	<0.001	0.426	0.127	0.010	0.615	<0.001	0.007	<0.001

Major axis shift

Neuropil	onega.sexmaculata	onega.gunilla	onega.anchiala	onega.illinissa	sexmaculata.gunilla	sexmaculata.anchiala	sexmaculata.illinissa	gunilla.anchiala	gunilla.illinissa	anchiala.illinissa
LOP	0.005	0.013	<0.001	0.251	0.668	<0.001	<0.001	<0.001	0.001	0.002

ii) *Napeogenes*

Test statistics

Slope shift (Neuropil ~ Rcbr*Species) — *Pairwise comparisons*

Neuropil	achaea.inachia	achaea.larina	achaea.pharo	achaea.sylphis	inachia.larina	inachia.pharo	inachia.sylphis	larina.pharo	larina.sylphis	pharo.sylphis
vLOB	5.652	10.840	8.189	6.875	0.139	0.481	0.726	0.364	0.911	0.111

Major axis shift (Neuropil ~ rCBR + Species, type = "shift")

Neuropil	achaea.inachia	achaea.larina	achaea.pharo	achaea.sylphis	inachia.larina	inachia.pharo	inachia.sylphis	larina.pharo	larina.sylphis	pharo.sylphis
ME	0.344	0.669	0.636	8.245	2.778	1.400	5.871	0.032	14.313	10.179
LOB	0.834	0.023	0.060	12.381	1.611	0.800	6.245	0.000	15.353	8.741
OL	0.417	0.514	0.486	8.627	2.488	1.291	5.490	0.024	14.485	10.135

***P values* (adjusted)**

Slope shift (Neuropil ~ rCBR*Species) — *Pairwise comparisons*

Neuropil	achaea.inachia	achaea.larina	achaea.pharo	achaea.sylphis	inachia.larina	inachia.pharo	inachia.sylphis	larina.pharo	larina.sylphis	pharo.sylphis
vLOB	0.161	0.010	0.041	0.084	1.000	0.999	0.993	1.000	0.984	1.000

Major axis shift (Neuropil ~ rCBR + Species, type = "shift") — *Pairwise comparisons*

Neuropil	achaea.inachia	achaea.larina	achaea.pharo	achaea.sylphis	inachia.larina	inachia.pharo	inachia.sylphis	larina.pharo	larina.sylphis	pharo.sylphis
ME	1.000	0.995	0.996	0.040	0.634	0.933	0.144	1.000	0.002	0.014
LOB	0.989	1.000	1.000	0.004	0.898	0.990	0.118	1.000	0.001	0.031
OL	0.999	0.998	0.999	0.033	0.704	0.948	0.176	1.000	0.001	0.014

***P values* (non-adjusted)**

Slope shift (Neuropil ~ rCBR*Species) — *Pairwise comparisons*

Neuropil	achaea.inachia	achaea.larina	achaea.pharo	achaea.sylphis	inachia.larina	inachia.pharo	inachia.sylphis	larina.pharo	larina.sylphis	pharo.sylphis
vLOB	0.017	0.001	0.004	0.009	0.709	0.488	0.394	0.546	0.340	0.739

Major axis shift (Neuropil ~ rCBR + Species, type = "shift") — *Pairwise comparisons*

Neuropil	achaea.inachia	achaea.larina	achaea.pharo	achaea.sylphis	inachia.larina	inachia.pharo	inachia.sylphis	larina.pharo	larina.sylphis	pharo.sylphis
ME	0.557	0.414	0.425	0.004	0.096	0.237	0.015	0.858	0.000	0.001
LOB	0.361	0.880	0.806	0.000	0.204	0.371	0.012	0.984	<0.001	0.003
OL	0.518	0.473	0.486	0.003	0.115	0.256	0.019	0.877	<0.001	0.001

iii) *Hypothyris*

Test statistics

Slope shift (Neuropil ~ rCBR*Species) — *Pairwise comparisons*

Neuropil	anastasia.fluonia	anastasia.moebiusi	anastasia.mamercus	fluonia.moebiusi	fluonia.mamercus	moebiusi.mamercus
LOP	3.203	6.494	1.842	2.092	3.917	5.621
AOTU	4.967	7.457	0.548	0.222	0.176	0.428

Elevation shift (Neuropil ~ rCBR + Species, type = "elevation")

Neuropil	anastasia.fluonia	anastasia.moebiusi	anastasia.mamercus	fluonia.moebiusi	fluonia.mamercus	moebiusi.mamercus
ME	3.915	8.316	0.595	32.895	0.175	10.067
LOB	0.596	10.453	1.410	10.907	3.079	2.686
OL	3.110	9.312	0.298	30.184	0.211	10.216

Major axis shift (Neuropil ~ rCBR + Species, type = "shift")

Neuropil	anastasia.fluonia	anastasia.moebiusi	anastasia.mamercus	fluonia.moebiusi	fluonia.mamercus	moebiusi.mamercus
AL	53.544	52.067	1.597	0.027	2.006	2.634

***P values* (adjusted)**

Slope shift (Neuropil ~ rCBR*Species) — *Pairwise comparisons*

Neuropil	anastasia.fluonia	anastasia.moebiusi	anastasia.mamercus	fluonia.moebiusi	fluonia.mamercus	moebiusi.mamercus
LOP	0.368	0.063	0.684	0.618	0.255	0.102
AOTU	0.145	0.037	0.975	0.998	0.999	0.987

Elevation shift (Neuropil ~ rCBR + Species, type = "elevation")

Neuropil	anastasia.fluonia	anastasia.moebiusi	anastasia.mamercus	fluonia.moebiusi	fluonia.mamercus	moebiusi.mamercus
ME	0.255	0.023	0.969	<0.001	0.999	0.009
LOB	0.969	0.007	0.800	0.006	0.391	0.473
OL	0.385	0.014	0.995	<0.001	0.998	0.008

Major axis shift (Neuropil ~ rCBR + Species, type = "shift")

Neuropil	anastasia.fluonia	anastasia.moebiusi	anastasia.mamercus	fluonia.moebiusi	fluonia.mamercus	moebiusi.mamercus
AL	<0.001	<0.001	0.750	1.000	0.640	0.485

***P values* (non-adjusted)**

Slope shift (Neuropil ~ rCBR*Species) — *Pairwise comparisons*

Neuropil	anastasia.fluonia	anastasia.moebiusi	anastasia.mamercus	fluonia.moebiusi	fluonia.mamercus	moebiusi.mamercus
LOP	0.074	0.011	0.175	0.148	0.048	0.018
vLOB	0.026	0.006	0.459	0.637	0.674	0.513

Elevation shift (Neuropil ~ rCBR + Species, type = "elevation")

Neuropil	anastasia.fluonia	anastasia.moebiusi	anastasia.mamercus	fluonia.moebiusi	fluonia.mamercus	moebiusi.mamercus
ME	0.048	0.004	0.440	<0.001	0.675	0.002
LOB	0.440	0.001	0.235	<0.001	0.079	0.101
OL	0.078	0.002	0.585	<0.001	0.646	0.001

Major axis shift (Neuropil ~ rCBR + Species, type = "shift")

Neuropil	anastasia.fluonia	anastasia.moebiusi	anastasia.mamercus	fluonia.moebiusi	fluonia.mamercus	moebiusi.mamercus
AL	<0.001	<0.001	0.206	0.869	0.157	0.105

UOB Open

Table S4: Principal Component Analyses

A) Monotypic Oleriina and Hypothyris, all neuropil

		PCA individual scores						
Species	Sex	PC1 (84.95% VAR)	PC2 (6.36% VAR)	PC3 (3.20% VAR)	PC4 (2.85% VAR)	PC5 (1.39% VAR)	PC6 (0.86% VAR)	PC7 (0.39% VAR)
Oleria_gunilla	Female	-1.709	-0.580	-0.354	-0.178	-0.530	-0.404	-0.133
Oleria_gunilla	Male	-2.483	0.729	-0.202	-0.386	0.182	0.269	0.024
Oleria_gunilla	Male	-2.268	0.017	1.015	-0.862	-0.166	0.753	0.115
Oleria_gunilla	Female	-1.347	-0.061	0.256	0.415	0.380	0.128	-0.099
Oleria_gunilla	Male	-1.482	0.305	0.331	-0.288	0.282	-0.048	0.121
Oleria_gunilla	Male	-4.315	1.196	0.095	-0.112	-0.182	-0.213	0.329
Oleria_gunilla	Female	-2.372	1.121	0.479	-0.400	-0.139	-0.152	-0.197
Oleria_onega	Female	0.702	0.416	0.390	0.207	0.416	0.427	-0.003
Oleria_onega	Female	-2.527	-0.022	-0.800	0.278	0.528	-0.106	-0.308
Oleria_onega	Male	0.221	-0.421	2.011	0.011	0.385	-0.561	0.006
Oleria_onega	Male	-0.998	-0.109	0.875	-0.184	0.391	-0.358	0.391
Oleria_sexmaculata	Female	-1.004	0.196	0.118	-0.246	-0.039	-0.224	0.150
Oleria_sexmaculata	Male	-1.213	-0.587	-0.245	-0.140	0.275	0.017	-0.111
Oleria_sexmaculata	Male	-2.675	0.169	-0.079	-0.403	0.360	-0.076	-0.077
Oleria_sexmaculata	Male	-1.646	-0.697	0.894	0.437	0.164	0.102	0.109
Oleria_sexmaculata	Male	-2.917	-1.254	0.698	0.798	0.162	0.088	0.035
Oleria_sexmaculata	Male	-1.683	-0.150	0.768	-0.540	-0.327	-0.354	0.215
Oleria_sexmaculata	Female	-4.492	0.664	0.393	-0.804	-0.200	-0.195	-0.483
Oleria_sexmaculata	Male	-1.117	0.268	0.681	-0.353	0.008	-0.279	0.016
Oleria_sexmaculata	Female	-2.094	0.064	0.268	0.162	-0.093	0.346	0.139
Oleria_assimilis	Male	-4.796	0.434	-0.310	-0.706	-0.043	0.114	-0.019
Oleria_assimilis	Male	-4.509	0.606	-0.360	-0.403	-0.621	0.354	0.012
Oleria_onega	Male	0.843	0.536	-0.150	0.129	0.120	0.126	0.138
Oleria_onega	Male	-0.295	1.009	-0.336	-0.422	-0.089	0.443	-0.224
Oleria_onega	Female	-1.775	0.818	1.114	0.956	0.798	0.292	0.109
Oleria_onega	Male	-4.625	0.017	0.292	0.436	0.724	0.334	0.176
Oleria_onega	Male	0.192	0.087	0.058	0.438	-0.495	0.362	-0.323
Oleria_onega	Female	-0.571	0.547	0.177	0.297	-0.440	-0.042	-0.157
Oleria_onega	Female	-0.115	-0.041	0.152	0.483	-0.104	0.091	-0.061
Oleria_onega	Female	-0.484	0.941	0.148	0.057	-0.286	-0.085	-0.198
Oleria_onega	Male	-0.415	-0.036	0.188	0.659	-0.084	-0.003	-0.111
Oleria_onega	Female	-0.654	-0.283	0.686	-1.341	-0.185	-0.030	-0.359
Oleria_onega	Female	-0.687	0.192	-0.152	-0.031	0.085	-0.132	-0.157
Oleria_onega	Female	-0.787	0.152	0.178	0.437	-0.367	0.000	-0.246
Oleria_onega	Male	-1.063	-0.155	-0.094	-0.097	-0.053	-0.122	-0.296
Oleria_onega	Female	-3.026	-0.033	-0.033	0.187	-0.187	-0.209	-0.079
Oleria_onega	Female	-1.737	-1.106	-1.000	0.536	0.061	-0.066	-0.231
Hyposcada_illinissa	Male	-1.300	-0.258	-0.017	-0.976	0.669	-0.167	-0.030
Hyposcada_illinissa	Male	0.012	0.431	-0.169	-0.715	0.579	0.470	0.103
Hyposcada_illinissa	Female	-2.375	1.263	-1.320	-0.086	0.438	-0.049	0.063
Hyposcada_illinissa	Female	-0.265	0.570	0.065	0.495	-0.135	0.061	-0.043
Hyposcada_illinissa	Female	-0.165	0.706	-0.653	1.107	0.190	-0.233	-0.023
Hyposcada_illinissa	Female	-1.282	0.350	-0.874	0.669	0.291	-0.057	-0.106
Hyposcada_illinissa	Female	-0.742	1.618	-0.217	0.817	-0.528	-0.234	0.520
Hyposcada_illinissa	Female	-0.599	0.312	-0.059	-0.097	0.276	0.000	-0.074
Hyposcada_kena	Female	-3.204	-1.028	0.145	0.123	0.088	-0.106	-0.054
Hyposcada_illinissa	Female	-0.600	0.897	-0.186	0.111	0.221	-0.002	0.040
Hyposcada_illinissa	Female	1.129	1.116	-0.256	-0.085	0.099	0.125	0.029
Hyposcada_kena	Male	-2.027	-1.665	-0.203	0.351	0.165	0.366	-0.109
Hypothyris_anastasia	Female	2.101	0.168	0.182	-0.113	-0.142	-0.409	-0.180
Hypothyris_anastasia	Male	3.495	-0.323	0.148	0.249	-0.301	-0.123	0.029
Hypothyris_anastasia	Male	2.919	-0.216	-0.777	-0.023	-0.108	0.206	0.130
Hypothyris_anastasia	Female	1.114	0.640	-0.377	-0.020	-0.352	-0.176	-0.234
Hypothyris_anastasia	Female	1.757	-0.111	-0.169	0.396	-0.508	-0.274	0.020
Hypothyris_anastasia	Male	-0.343	-1.193	0.004	0.107	-0.638	0.874	-0.110
Hypothyris_anastasia	Male	0.714	-2.556	-0.442	0.077	0.230	-0.204	0.202
Hypothyris_anastasia	Male	4.063	0.603	-0.429	0.059	-0.063	-0.304	-0.143
Hypothyris_anastasia	Female	2.612	0.030	-0.062	0.476	-0.538	-0.069	-0.067
Hypothyris_anastasia	Male	5.123	0.033	0.305	0.046	-0.013	-0.084	-0.122
Hypothyris_anastasia	Male	4.037	0.210	-0.033	0.198	0.098	0.077	0.121
Hypothyris_anastasia	Male	1.724	0.563	0.387	0.269	0.096	0.092	-0.144
Hypothyris_anastasia	Male	3.834	0.153	-0.005	-0.068	0.237	0.156	-0.104
Hypothyris_anastasia	Male	3.119	-0.217	-0.698	0.122	0.157	0.229	0.065
Hypothyris_anastasia	Female	3.810	0.508	0.288	0.970	-0.018	-0.082	-0.060
Hypothyris_anastasia	Male	4.098	0.018	-0.235	-0.471	0.356	-0.226	0.084
Hypothyris_anastasia	Male	4.700	0.685	-0.012	-0.360	0.052	-0.113	0.111
Hypothyris_anastasia	Male	5.155	0.584	0.152	-0.102	-0.124	0.172	-0.020
Hypothyris_anastasia	Male	0.792	-0.106	0.434	-0.207	-0.133	-0.223	-0.065
Hypothyris_anastasia	Male	4.455	-0.303	0.361	0.070	-0.035	0.108	-0.237
Hypothyris_anastasia	Female	0.912	-0.417	-0.208	0.440	0.135	-0.007	-0.104
Hypothyris_anastasia	Male	4.218	0.230	-0.044	-0.014	0.468	-0.182	0.000
Hypothyris_anastasia	Female	4.312	0.395	-0.002	0.576	0.290	0.346	-0.091
Hypothyris_anastasia	Female	4.487	0.941	0.070	0.231	0.262	0.288	0.122
Hypothyris_anastasia	Male	4.576	-0.994	0.214	-0.001	0.251	0.062	-0.348
Hypothyris_anastasia	Female	2.905	-0.157	0.405	0.048	-0.074	0.166	0.076
Hypothyris_anastasia	Female	3.450	-0.661	-0.706	-0.929	0.389	0.085	0.106
Hypothyris_fluonia	Female	-0.611	-0.328	-0.215	0.036	-0.210	0.055	-0.115
Hypothyris_fluonia	Male	3.652	0.466	-0.508	-0.785	-0.321	-0.425	0.189
Hypothyris_fluonia	Female	-4.806	0.184	-0.763	0.317	-0.018	-0.051	0.111
Hypothyris_fluonia	Female	0.769	-0.621	-0.845	0.110	-0.102	0.069	0.022
Hypothyris_fluonia	Male	0.008	-0.948	-0.727	-0.260	0.154	-0.117	0.072
Hypothyris_anastasia	Female	2.245	-0.132	0.269	0.118	-0.196	-0.050	0.043
Hypothyris_fluonia	Female	-4.122	-0.993	-0.301	0.151	0.006	0.017	0.066
Hypothyris_fluonia	Male	1.471	-0.497	-0.478	-0.544	-0.079	-0.235	-0.037
Hypothyris_fluonia	Male	1.936	-0.555	-0.140	-0.305	-0.065	0.157	0.099
Hypothyris_fluonia	Female	-0.952	0.102	-0.143	0.643	-0.120	0.010	0.223
Hypothyris_fluonia	Female	-1.310	0.354	-0.064	0.500	0.022	0.215	-0.046
Hypothyris_fluonia	Male	-0.295	-0.795	-0.082	-0.213	0.106	-0.444	0.168
Hypothyris_fluonia	Female	-1.270	-0.977	0.055	0.550	-0.198	-0.270	0.058
Hypothyris_fluonia	Female	-1.657	-0.656	-0.123	0.291	-0.180	0.135	0.295
Hypothyris_fluonia	Male	-0.055	-0.789	-0.204	-0.234	-0.269	-0.321	0.137
Hypothyris_fluonia	Male	-1.593	-0.835	-0.420	-0.446	0.108	0.014	-0.107
Hypothyris_fluonia	Male	-1.239	-0.916	-0.298	-0.095	0.108	0.139	0.102
Hypothyris_fluonia	Female	-1.962	-0.113	-0.389	0.271	-0.121	-0.091	0.090
Hypothyris_fluonia	Male	-0.198	0.197	-0.300	-0.388	-0.118	-0.125	0.239
Hypothyris_fluonia	Female	0.588	0.176	0.042	0.249	-0.111	-0.209	0.338
Hypothyris_fluonia	Male	1.160	-0.510	-0.153	-0.021	0.035	-0.253	0.078
Hypothyris_fluonia	Female	0.103	0.145	-0.237	-0.268	0.396	-0.074	-0.013
Hypothyris_mamercus	Female	3.237	0.094	0.418	-0.217	-0.147	-0.048	0.044
Hypothyris_moebiusi	Male	1.612	0.427	0.321	-0.158	-0.440	0.446	0.163
Hypothyris_anastasia	Male	-1.538	0.111	-0.101	-0.582	-0.177	0.065	0.163
Hypothyris_moebiusi	Female	-1.823	0.026	0.367	0.230	0.050	-0.387	-0.094
Hypothyris_moebiusi	Male	-0.953	-0.469	0.628	0.416	0.062	-0.013	-0.078
Hypothyris_moebiusi	Male	0.903	0.088	-0.075	-0.974	0.595	0.183	0.122
Hypothyris_moebiusi	Male	-0.639	1.047	0.175	-0.613	-0.180	0.165	-0.006
Hypothyris_moebiusi	Male	-0.649	-0.306	-0.024	-0.353	0.147	0.270	-0.015
Hypothyris_moebiusi	Male	-1.774	0.659	-0.256	-0.211	-0.006	-0.194	-0.210
Hypothyris_moebiusi	Female	-2.906	0.419	0.665	0.472	0.155	-0.368	-0.189
Hypothyris_mamercus	Male	2.868	-0.863	0.456	-0.052	-0.059	0.132	0.166
Hypothyris_mamercus	Female	0.867	-1.291	0.645	-0.522	-0.942	0.286	0.115
Hypothyris_mamercus	Female	-1.859	0.331	-0.356	0.119	-0.901	0.171	0.238

	Loadings						
Neuropil	PC1 (84.95% VAR)	PC2 (6.36% VAR)	PC3 (3.20% VAR)	PC4 (2.85% VAR)	PC5 (1.39% VAR)	PC6 (0.86% VAR)	PC7 (0.39% VAR)
rCBR	0.385	0.150	0.379	0.382	0.644	0.296	0.190
ME	0.395	-0.271	-0.215	-	-	-0.499	0.689
LOP	0.385	-0.181	-0.558	-	-0.204	0.680	-
LOB	0.394	-0.264	-0.242	0.105	0.289	-0.396	-0.682
AL	0.381	0.248	0.325	0.467	-0.663	-0.110	-0.136
AOTU	0.365	-0.366	0.538	-0.634	-0.120	0.147	-
aME	0.337	0.779	-0.210	-0.469	-	-0.102	-

B) *All individuals, all neuropil*

		PCA individual scores						
Species	Sex	PC1 (81.85% var)	PC2 (7.26% VAR)	PC3 (3.85% VAR)	PC4 (3.29% VAR)	PC5 (1.95% VAR)	PC6 (1.16% VAR)	PC7 (0.65% VAR)
Napeogenes rhezia	Female	-2.079	-1.156	0.070	0.281	-0.008	0.177	-0.098
Napeogenes rhezia	Female	0.276	1.139	0.567	0.502	0.048	0.378	-0.079
Napeogenes inachia	Male	-0.580	0.589	2.122	0.181	0.162	-0.950	0.038
Napeogenes inachia	Male	-0.954	-1.034	-0.579	0.188	0.008	0.826	0.269
Napeogenes larina	Female	0.874	0.040	-0.240	0.021	0.128	0.160	-0.098
Napeogenes larina	Female	-1.314	0.617	0.095	0.567	0.433	0.117	-0.227
Napeogenes larina	Male	3.196	0.771	0.217	-0.506	-0.213	0.232	0.259
Napeogenes larina	Female	-1.762	1.167	-0.640	0.999	-0.368	-0.073	0.156
Napeogenes larina	Male	1.867	-0.385	1.614	0.688	0.584	-0.167	0.077
Napeogenes larina	Male	0.292	-1.085	0.249	0.331	-0.056	-0.161	0.323
Napeogenes larina	Female	1.941	1.004	-0.366	0.004	0.155	-0.041	0.263
Napeogenes larina	Male	2.736	1.482	-0.170	-0.123	0.003	-0.084	0.127
Napeogenes larina	Male	0.228	0.073	0.365	-0.347	0.240	0.030	0.102
Napeogenes larina	Female	-0.810	0.349	0.102	0.190	-0.124	0.148	-0.023
Napeogenes larina	Female	0.698	0.964	-0.376	-0.757	-0.674	-0.625	-0.097
Napeogenes larina	Male	-1.184	-0.274	-0.507	0.083	0.503	0.324	-0.012
Napeogenes larina	Female	-0.450	1.285	-0.154	0.120	-0.597	-0.004	0.177
Napeogenes pharo	Male	-1.508	-0.504	0.025	0.148	0.084	0.089	0.316
Napeogenes pharo	Male	0.880	1.021	-1.006	-0.875	-1.091	-0.578	0.039
Napeogenes pharo	Male	1.291	-0.427	-0.440	0.218	-0.268	0.008	0.041
Napeogenes pharo	Female	-0.201	0.944	0.424	0.066	-0.023	-0.166	0.162
Napeogenes pharo	Female	1.209	0.928	0.564	-0.307	-0.501	-0.109	-0.149
Napeogenes pharo	Male	2.445	1.116	0.106	-0.405	0.421	0.256	0.052
Napeogenes sylphis	Male	-3.246	1.263	0.985	0.708	-0.005	0.144	0.532
Napeogenes rhezia	Male	0.162	0.852	0.144	-0.916	-0.271	0.064	-0.272
Napeogenes rhezia	Male	0.284	0.678	-0.122	0.019	-0.075	0.359	-0.276
Napeogenes rhezia	Male	0.382	-0.420	0.223	-0.165	-0.504	-0.114	-0.234
Napeogenes rhezia	Female	0.469	0.558	-0.573	0.544	-0.162	-0.097	-0.088
Napeogenes inachia	Male	-1.512	-0.711	0.306	-0.572	-0.558	0.393	-0.351
Napeogenes inachia	Male	1.045	-0.256	0.196	-0.277	-0.327	-0.174	0.000
Napeogenes inachia	Male	0.507	0.183	0.140	-0.813	-0.614	-0.106	-0.059
Napeogenes inachia	Female	-0.806	0.013	0.183	0.534	-0.649	-0.375	0.182
Napeogenes sylphis	Male	-0.181	0.563	0.546	-0.354	-0.160	0.147	-0.209
Napeogenes sylphis	Male	0.655	0.347	0.082	-0.214	-0.233	0.563	0.415
Napeogenes sylphis	Female	-4.114	0.418	0.269	0.013	0.058	-0.373	-0.225
Napeogenes sylphis	Female	-1.940	0.567	-0.188	0.082	-0.328	0.349	-0.120
Napeogenes sylphis	Male	-3.444	0.742	-0.667	-0.396	0.081	-0.393	-0.397
Napeogenes sylphis	Female	-3.471	1.241	-0.614	0.464	-0.215	0.374	-0.095
Napeogenes sylphis	Male	1.305	0.219	0.221	-0.002	-0.226	0.124	0.094
Napeogenes sylphis	Female	-3.838	0.901	-0.441	-0.745	0.131	0.084	-0.812
Napeogenes sylphis	Male	-3.637	0.797	-0.095	-0.294	-0.306	0.226	-0.015
Napeogenes sylphis	Male	-2.552	0.458	-0.105	0.117	-0.203	-0.259	-0.145
Napeogenes sylphis	Male	-2.284	-0.451	0.313	0.012	-0.262	0.017	-0.268
Oleria gunilla	Female	-1.811	-0.632	-0.414	-0.400	-0.445	-0.516	0.022
Oleria gunilla	Male	-2.673	0.665	-0.198	-0.286	0.400	0.269	0.033
Oleria gunilla	Male	-2.436	-0.002	0.943	-1.001	-0.019	0.903	0.030
Oleria gunilla	Female	-1.430	-0.290	0.278	0.415	0.378	0.146	-0.165
Oleria gunilla	Male	-1.594	0.193	0.383	-0.228	0.415	0.024	0.193
Oleria gunilla	Male	-4.633	1.122	0.212	0.042	-0.038	-0.136	0.420
Oleria gunilla	Female	-2.561	1.088	0.581	-0.319	0.043	-0.157	-0.116
Oleria onega	Female	0.750	0.233	0.383	0.302	0.459	0.518	-0.131
Oleria onega	Female	-2.698	-0.218	-0.787	0.318	0.660	-0.267	-0.266
Oleria onega	Male	0.245	-0.648	2.186	-0.066	0.296	-0.358	0.127
Oleria onega	Male	-1.067	-0.276	0.982	-0.145	0.433	-0.171	0.530
Oleria sexmaculata	Female	-1.076	0.115	0.143	-0.237	0.077	-0.180	0.262
Oleria sexmaculata	Male	-1.286	-0.726	-0.296	-0.239	0.383	-0.038	-0.056
Oleria sexmaculata	Male	-2.871	0.070	-0.039	-0.373	0.558	-0.108	0.028
Oleria sexmaculata	Male	-1.736	-0.950	0.906	0.293	0.053	0.223	0.040
Oleria sexmaculata	Male	-3.079	-1.577	0.687	0.554	-0.025	0.154	-0.065
Oleria sexmaculata	Male	-1.800	-0.194	0.808	-0.660	-0.241	-0.250	0.384
Oleria sexmaculata	Female	-4.829	0.676	0.478	-0.866	0.059	-0.311	-0.333
Oleria sexmaculata	Male	-1.201	0.185	0.756	-0.360	0.113	-0.212	0.137
Oleria sexmaculata	Female	-2.231	-0.085	0.234	0.111	-0.076	0.418	0.047
Oleria assimilis	Male	-5.147	0.422	-0.308	-0.716	0.227	0.057	0.080
Oleria assimilis	Male	-4.832	0.616	-0.426	-0.477	-0.431	0.302	-0.006
Oleria onega	Male	0.900	0.412	-0.169	0.239	0.204	0.180	0.110
Oleria onega	Male	-0.336	1.005	-0.395	-0.335	0.148	0.386	-0.275
Oleria onega	Female	-1.898	0.466	1.275	1.175	0.690	0.484	-0.091
Oleria onega	Male	-4.941	-0.269	0.361	0.505	0.738	0.420	0.077
Oleria onega	Male	0.222	-0.038	-0.043	0.298	-0.524	0.304	-0.480
Oleria onega	Female	-0.606	0.443	0.178	0.267	-0.425	-0.060	-0.191
Oleria onega	Female	-0.107	-0.223	0.121	0.424	-0.143	0.105	-0.136
Oleria onega	Female	-0.526	0.870	0.184	0.115	-0.190	-0.111	-0.191
Oleria onega	Male	-0.424	-0.247	0.181	0.602	-0.154	-0.003	-0.186
Oleria onega	Female	-0.714	-0.215	0.649	-1.546	0.080	-0.066	-0.183
Oleria onega	Female	-0.734	0.081	-0.146	-0.025	0.199	-0.184	-0.098
Oleria onega	Female	-0.829	0.008	0.159	0.341	-0.391	-0.044	-0.305
Oleria onega	Male	-1.131	-0.255	-0.115	-0.197	0.050	-0.215	-0.235
Oleria onega	Female	-3.227	-0.172	-0.012	0.102	-0.146	-0.258	-0.027
Oleria onega	Female	-1.823	-1.317	-1.115	0.336	0.067	-0.246	-0.223
Hyposcada anchiala	Male	-0.829	-1.762	0.384	0.621	-0.136	-0.252	0.047
Hyposcada anchiala	Male	3.173	0.737	-0.094	0.671	0.050	-0.198	-0.045
Hyposcada anchiala	Male	-1.206	-0.576	-0.375	-0.278	0.283	-0.135	-0.231
Hyposcada anchiala	Female	1.450	0.018	-0.700	0.911	0.228	-0.366	-0.074
Hyposcada anchiala	Male	0.932	-0.937	-0.578	0.381	0.230	-0.634	-0.195
Hyposcada anchiala	Male	4.624	0.363	0.942	-1.347	0.360	-0.511	-0.797
Hyposcada anchiala	Male	1.540	-0.534	-0.256	0.099	0.118	-0.232	-0.055
Hyposcada anchiala	Female	2.414	-0.029	-0.454	0.384	-0.108	0.115	-0.297
Hyposcada anchiala	Male	1.627	-0.302	-0.102	0.257	0.282	-0.125	0.160
Hyposcada anchiala	Female	3.091	-0.387	0.677	0.093	0.150	-0.006	-0.264
Hyposcada anchiala	Male	-0.836	-0.074	-0.478	0.310	-0.018	0.126	-0.019
Hyposcada anchiala	Female	1.042	-0.929	-0.146	0.889	0.305	-0.215	-0.014
Hyposcada illinissa	Male	-1.406	-0.314	0.007	-0.960	0.957	-0.178	0.184
Hyposcada illinissa	Male	-0.007	0.377	-0.214	-0.585	0.848	0.524	0.110
Hyposcada illinissa	Female	-2.563	1.181	-1.283	0.211	0.729	-0.151	0.146
Hyposcada illinissa	Female	-0.278	0.411	0.072	0.541	-0.137	0.076	-0.117
Hyposcada illinissa	Female	-0.164	0.451	-0.605	1.283	0.153	-0.284	-0.075
Hyposcada illinissa	Female	-1.362	0.140	-0.874	0.774	0.348	-0.160	-0.133
Hyposcada illinissa	Female	-0.795	1.483	-0.140	1.066	-0.555	-0.111	0.486
Hyposcada illinissa	Female	-0.645	0.192	-0.044	-0.037	0.408	-0.009	-0.038
Hyposcada kena	Female	-3.402	-1.222	0.123	-0.096	0.087	-0.135	-0.005
Hyposcada illinissa	Female	-0.652	0.773	-0.142	0.284	0.347	0.014	0.048
Hyposcada illinissa	Female	1.192	1.049	-0.255	0.111	0.267	0.148	0.021
Hyposcada kena	Male	-2.127	-1.904	-0.356	0.038	0.112	0.310	-0.198
Hypothyris anastasia	Female	2.249	0.096	0.191	-0.132	-0.060	-0.429	-0.060
Hypothyris anastasia	Male	3.759	-0.442	0.064	0.151	-0.338	-0.079	0.034
Hypothyris anastasia	Male	3.136	-0.289	-0.942	-0.050	-0.008	0.193	0.119
Hypothyris anastasia	Female	1.188	0.602	-0.408	0.003	-0.225	-0.264	-0.182
Hypothyris anastasia	Female	1.901	-0.223	-0.232	0.304	-0.540	-0.285	0.051
Hypothyris anastasia	Male	-0.330	-1.300	-0.265	-0.281	-0.688	0.858	-0.332
Hypothyris anastasia	Male	4.344	0.539	-0.458	0.168	0.052	-0.350	-0.064
Hypothyris anastasia	Female	2.814	-0.085	-0.148	0.390	-0.588	-0.075	-0.111
Hypothyris anastasia	Male	5.488	-0.068	0.247	0.043	0.012	-0.038	-0.114
Hypothyris anastasia	Male	4.325	0.082	-0.097	0.272	0.129	0.156	0.088
Hypothyris anastasia	Male	1.844	0.416	0.399	0.340	0.121	0.134	-0.209
Hypothyris anastasia	Male	4.102	0.049	-0.075	-0.018	0.332	0.182	-0.126
Hypothyris anastasia	Male	3.349	-0.337	-0.839	0.134	0.240	0.217	0.030
Hypothyris anastasia	Female	4.092	0.281	0.295	1.064	-0.139	-0.018	-0.174
Hypothyris anastasia	Male	4.378	-0.038	-0.279	-0.384	0.538	-0.192	0.232
Hypothyris anastasia	Male	5.015	0.660	-0.043	-0.213	0.211	-0.037	0.192
Hypothyris anastasia	Male	5.511	0.536	0.072	-0.025	-0.040	0.243	-0.061
Hypothyris anastasia	Male	0.852	-0.190	0.431	-0.286	-0.069	-0.194	0.023
Hypothyris anastasia	Male	4.779	-0.420	0.265	-0.022	-0.032	0.128	-0.281
Hypothyris anastasia	Female	0.997	-0.611	-0.266	0.376	0.123	-0.038	-0.131
Hypothyris anastasia	Male	4.510	0.100	-0.050	0.120	0.568	-0.138	0.067
Hypothyris anastasia	Female	4.622	0.202	-0.069	0.689	0.275	0.396	-0.251
Hypothyris anastasia	Female	4.793	0.814	0.038	0.451	0.325	0.404	0.020
Hypothyris anastasia	Male	4.917	-1.144	0.091	-0.170	0.262	0.034	-0.356
Hypothyris anastasia	Female	3.119	-0.275	0.329	-0.006	-0.073	0.263	0.030
Hypothyris anastasia	Female	3.689	-0.665	-0.865	-0.962	0.659	0.071	0.259
Hypothyris fluonia	Female	-0.640	-0.435	-0.296	-0.096	-0.152	0.002	-0.110
Hypothyris fluonia	Male	3.894	0.542	-0.569	-0.718	-0.073	-0.416	0.422
Hypothyris fluonia	Female	-5.132	0.025	-0.758	0.328	0.074	-0.125	0.139
Hypothyris fluonia	Female	0.844	-0.734	-0.992	-0.004	-0.031	-0.005	0.039
Hypothyris fluonia	Male	0.026	-1.051	-0.840	-0.391	0.285	-0.180	0.196
Hypothyris anastasia	Female	2.415	-0.247	0.215	0.053	-0.197	0.009	0.046
Hypothyris fluonia	Female	-4.383	-1.176	-0.362	-0.057	0.030	-0.030	0.097
Hypothyris fluonia	Male	1.579	-0.519	-0.573	-0.649	0.098	-0.296	0.129
Hypothyris fluonia	Male	2.083	-0.625	-0.271	-0.419	0.027	0.187	0.127
Hypothyris fluonia	Female	-1.000	-0.095	-0.160	0.649	-0.168	0.060	0.165
Hypothyris fluonia	Female	-1.393	0.170	-0.075	0.526	0.029	0.216	-0.148
Hypothyris fluonia	Male	-0.300	-0.916	-0.099	-0.325	0.181	-0.429	0.353
Hypothyris fluonia	Female	-1.323	-1.193	0.015	0.339	-0.297	-0.274	0.088
Hypothyris fluonia	Female	-1.746	-0.825	-0.208	0.147	-0.208	0.191	0.264
Hypothyris fluonia	Male	-0.039	-0.864	-0.279	-0.412	-0.201	-0.330	0.287
Hypothyris fluonia	Male	-1.693	-0.919	-0.513	-0.619	0.267	-0.071	-0.005
Hypothyris fluonia	Male	-1.305	-1.057	-0.402	-0.256	0.174	0.130	0.128
Hypothyris fluonia	Female	-2.084	-0.259	-0.418	0.221	-0.081	-0.118	0.116
Hypothyris fluonia	Male	-0.215	0.160	-0.334	-0.369	0.045	-0.094	0.355
Hypothyris fluonia	Female	0.639	0.038	0.044	0.289	-0.104	-0.109	0.383
Hypothyris fluonia	Male	1.256	-0.632	-0.202	-0.097	0.089	-0.247	0.178
Hypothyris fluonia	Female	0.105	0.040	-0.236	-0.198	0.566	-0.084	0.075
Hypothyris moebiusi	Male	1.725	0.388	0.220	-0.186	-0.381	0.556	0.064
Hypothyris anastasia	Male	-1.651	0.093	-0.147	-0.624	0.007	0.091	0.251
Hypothyris moebiusi	Female	-1.942	-0.146	0.446	0.198	0.066	-0.390	-0.012
Hypothyris moebiusi	Male	-0.999	-0.689	0.636	0.290	-0.011	0.033	-0.120
Hypothyris moebiusi	Male	0.947	0.061	-0.112	-0.888	0.885	0.239	0.239
Hypothyris moebiusi	Male	-0.707	1.061	0.181	-0.523	0.044	0.200	0.031
Hypothyris moebiusi	Male	-0.690	-0.399	-0.099	-0.432	0.279	0.277	-0.009
Hypothyris moebiusi	Male	-1.910	0.596	-0.216	-0.153	0.178	-0.278	-0.115
Hypothyris moebiusi	Female	-3.105	0.200	0.813	0.499	0.137	-0.360	-0.155
Hypothyris mamercus	Female	3.465	0.031	0.372	-0.239	-0.085	0.035	0.082
Hypothyris mamercus	Male	3.091	-0.994	0.342	-0.216	-0.084	0.245	0.153
Hypothyris mamercus	Female	0.957	-1.294	0.437	-0.954	-0.956	0.366	0.110
Hypothyris mamercus	Female	-1.977	0.296	-0.457	0.016	-0.861	0.183	0.198
Napeogenes larina	Female	0.552	-2.311	0.305	0.252	-0.947	-0.013	-0.010
Napeogenes larina	Male	0.722	0.260	0.244	-0.031	-0.886	0.436	0.309
Napeogenes larina	Male	0.606	0.254	0.123	-0.180	-1.048	0.317	0.289
Napeogenes larina	Female	0.358	-0.249	0.274	0.674	-0.553	-0.418	0.002

	Loadings						
Neuropil	PC1 (81.85% var)	PC2 (7.26% VAR)	PC3 (3.85% VAR)	PC4 (3.29% VAR)	PC5 (1.95% VAR)	PC6 (1.16% VAR)	PC7 (0.65% VAR)
rCBR	0.385	-	0.401	0.493	0.537	0.393	-
ME	0.401	-0.258	-0.172	-	-	-0.340	0.788
LOP	0.383	-0.142	-0.655	-	-0.179	0.593	-0.139
LOB	0.396	-0.279	-0.186	-	0.300	-0.568	-0.562
AL	0.384	0.229	0.302	0.356	-0.732	-0.113	-0.186
AOTU	0.368	-0.274	0.478	-0.725	-	0.172	-
aME	0.325	0.841	-0.162	-0.310	0.216	-0.116	-

UOB Open

Table S5: Discriminant Function Analyses

A) *Monotypic Oleriina and Hypothyris* (Clade ~ ME + LOP + LOB + aME + AL + AOTU + rCBR)

Discriminant Function Coefficients (2 GROUPS: CLADE)

Neuropil	DF1
rCBR	-5.440
ME	15.458
LOP	4.602
LOB	-6.733
aME	-1.739
AL	1.394
AOTU	0.450

DFA Predictions

Clade	Posterior probabilities: *Oleriina*	Posterior probabilities: *Hypothyris*	*Final Prediction*
Oleriina	0.217	0.783	*Hypothyris*
Oleriina	0.938	0.062	*Oleriina*
Oleriina	0.917	0.083	*Oleriina*
Oleriina	0.903	0.097	*Oleriina*
Oleriina	0.839	0.161	*Oleriina*
Oleriina	0.956	0.044	*Oleriina*
Oleriina	0.978	0.022	*Oleriina*
Oleriina	0.792	0.208	*Oleriina*
Oleriina	0.936	0.064	*Oleriina*
Oleriina	0.847	0.153	*Oleriina*
Oleriina	0.607	0.393	*Oleriina*
Oleriina	0.487	0.513	*Hypothyris*
Oleriina	0.591	0.409	*Oleriina*
Oleriina	0.937	0.063	*Oleriina*
Oleriina	0.776	0.224	*Oleriina*
Oleriina	0.831	0.169	*Oleriina*
Oleriina	0.467	0.533	*Hypothyris*
Oleriina	0.996	0.004	*Oleriina*
Oleriina	0.795	0.205	*Oleriina*
Oleriina	0.792	0.208	*Oleriina*
Oleriina	0.972	0.028	*Oleriina*
Oleriina	0.921	0.079	*Oleriina*
Oleriina	0.337	0.663	*Hypothyris*
Oleriina	0.835	0.165	*Oleriina*
Oleriina	0.995	0.005	*Oleriina*
Oleriina	0.993	0.007	*Oleriina*
Oleriina	0.536	0.464	*Oleriina*
Oleriina	0.668	0.332	*Oleriina*
Oleriina	0.500	0.500	*Oleriina*
Oleriina	0.816	0.184	*Oleriina*
Oleriina	0.617	0.383	*Oleriina*
Oleriina	0.756	0.244	*Oleriina*
Oleriina	0.691	0.309	*Oleriina*
Oleriina	0.704	0.296	*Oleriina*
Oleriina	0.713	0.287	*Oleriina*
Oleriina	0.845	0.155	*Oleriina*
Oleriina	0.309	0.691	*Hypothyris*
Oleriina	0.793	0.207	*Oleriina*
Oleriina	0.724	0.276	*Oleriina*
Oleriina	0.893	0.107	*Oleriina*
Oleriina	0.666	0.334	*Oleriina*
Oleriina	0.592	0.408	*Oleriina*
Oleriina	0.739	0.261	*Oleriina*
Oleriina	0.308	0.692	*Hypothyris*
Oleriina	0.769	0.231	*Oleriina*
Oleriina	0.779	0.221	*Oleriina*
Oleriina	0.798	0.202	*Oleriina*
Oleriina	0.508	0.492	*Oleriina*
Oleriina	0.448	0.552	*Hypothyris*
Hypothyris	0.157	0.843	*Hypothyris*
Hypothyris	0.014	0.986	*Hypothyris*
Hypothyris	0.010	0.990	*Hypothyris*
Hypothyris	0.272	0.728	*Hypothyris*
Hypothyris	0.031	0.969	*Hypothyris*
Hypothyris	0.126	0.874	*Hypothyris*
Hypothyris	0.004	0.996	*Hypothyris*
Hypothyris	0.036	0.964	*Hypothyris*
Hypothyris	0.033	0.967	*Hypothyris*
Hypothyris	0.022	0.978	*Hypothyris*
Hypothyris	0.028	0.972	*Hypothyris*
Hypothyris	0.572	0.428	*Oleriina*
Hypothyris	0.087	0.913	*Hypothyris*
Hypothyris	0.021	0.979	*Hypothyris*
Hypothyris	0.109	0.891	*Hypothyris*
Hypothyris	0.020	0.980	*Hypothyris*
Hypothyris	0.023	0.977	*Hypothyris*
Hypothyris	0.025	0.975	*Hypothyris*
Hypothyris	0.283	0.717	*Hypothyris*
Hypothyris	0.043	0.957	*Hypothyris*
Hypothyris	0.222	0.778	*Hypothyris*
Hypothyris	0.059	0.941	*Hypothyris*
Hypothyris	0.116	0.884	*Hypothyris*
Hypothyris	0.093	0.907	*Hypothyris*
Hypothyris	0.032	0.968	*Hypothyris*
Hypothyris	0.061	0.939	*Hypothyris*
Hypothyris	0.009	0.991	*Hypothyris*
Hypothyris	0.348	0.652	*Hypothyris*
Hypothyris	0.005	0.995	*Hypothyris*
Hypothyris	0.911	0.089	*Oleriina*
Hypothyris	0.040	0.960	*Hypothyris*
Hypothyris	0.060	0.940	*Hypothyris*
Hypothyris	0.067	0.933	*Hypothyris*
Hypothyris	0.729	0.271	*Oleriina*
Hypothyris	0.037	0.963	*Hypothyris*
Hypothyris	0.034	0.966	*Hypothyris*
Hypothyris	0.361	0.639	*Hypothyris*
Hypothyris	0.822	0.178	*Oleriina*
Hypothyris	0.103	0.897	*Hypothyris*
Hypothyris	0.204	0.796	*Hypothyris*
Hypothyris	0.204	0.796	*Hypothyris*
Hypothyris	0.044	0.956	*Hypothyris*
Hypothyris	0.421	0.579	*Hypothyris*
Hypothyris	0.249	0.751	*Hypothyris*
Hypothyris	0.482	0.518	*Oleriina*
Hypothyris	0.159	0.841	*Hypothyris*
Hypothyris	0.106	0.894	*Hypothyris*
Hypothyris	0.065	0.935	*Hypothyris*
Hypothyris	0.527	0.473	*Oleriina*
Hypothyris	0.051	0.949	*Hypothyris*
Hypothyris	0.133	0.867	*Hypothyris*
Hypothyris	0.453	0.547	*Hypothyris*
Hypothyris	0.848	0.152	*Oleriina*
Hypothyris	0.731	0.269	*Oleriina*
Hypothyris	0.394	0.606	*Hypothyris*
Hypothyris	0.799	0.201	*Oleriina*
Hypothyris	0.534	0.466	*Oleriina*
Hypothyris	0.890	0.110	*Oleriina*
Hypothyris	0.984	0.016	*Oleriina*
Hypothyris	0.019	0.981	*Hypothyris*
Hypothyris	0.014	0.986	*Hypothyris*

% Correct

Oleriina	*Hypothyris*
85.714	80.328

B) ***All individuals*** (Clade ~ ME + LOP + LOB + aME + AL + AOTU + rCBR)

Discriminant Function Coefficients (3 GROUPS: CLADE)

Neuropil	DF1	DF2
rCBR	-1.910	8.510
ME	13.392	-2.341
LOP	4.687	-3.895
LOB	-4.771	12.714
aME	-0.571	-0.766
AL	-4.382	-12.234
AOTU	1.515	8.510

DFA Predictions

Clade	Posterior probabilities: *Oleriina*	Posterior probabilities: *Napeogenes*	Posterior probabilities: *Hypothyris*	*Final prediction*
Napeogenes	0.634	0.128	0.238	*Oleriina*
Napeogenes	0.349	0.574	0.077	*Napeogenes*
Napeogenes	0.705	0.270	0.025	*Oleriina*
Napeogenes	0.172	0.098	0.731	*Hypothyris*
Napeogenes	0.353	0.194	0.453	*Hypothyris*
Napeogenes	0.800	0.143	0.057	*Oleriina*
Napeogenes	0.051	0.388	0.560	*Hypothyris*
Napeogenes	0.213	0.701	0.086	*Napeogenes*
Napeogenes	0.744	0.074	0.183	*Oleriina*
Napeogenes	0.231	0.101	0.668	*Hypothyris*
Napeogenes	0.164	0.268	0.568	*Hypothyris*
Napeogenes	0.127	0.482	0.390	*Napeogenes*
Napeogenes	0.420	0.208	0.372	*Oleriina*
Napeogenes	0.368	0.461	0.171	*Napeogenes*
Napeogenes	0.074	0.742	0.184	*Napeogenes*
Napeogenes	0.607	0.072	0.321	*Oleriina*
Napeogenes	0.064	0.856	0.080	*Napeogenes*
Napeogenes	0.408	0.174	0.417	*Hypothyris*
Napeogenes	0.017	0.774	0.209	*Napeogenes*
Napeogenes	0.141	0.187	0.672	*Hypothyris*
Napeogenes	0.317	0.536	0.147	*Napeogenes*
Napeogenes	0.105	0.799	0.097	*Napeogenes*
Napeogenes	0.270	0.266	0.464	*Hypothyris*
Napeogenes	0.318	0.655	0.027	*Napeogenes*
Napeogenes	0.189	0.676	0.135	*Napeogenes*
Napeogenes	0.332	0.496	0.172	*Napeogenes*
Napeogenes	0.237	0.469	0.294	*Napeogenes*
Napeogenes	0.303	0.400	0.297	*Napeogenes*
Napeogenes	0.273	0.557	0.170	*Napeogenes*
Napeogenes	0.184	0.340	0.475	*Hypothyris*
Napeogenes	0.093	0.643	0.265	*Napeogenes*
Napeogenes	0.171	0.648	0.180	*Napeogenes*
Napeogenes	0.312	0.578	0.110	*Napeogenes*
Napeogenes	0.083	0.439	0.479	*Hypothyris*
Napeogenes	0.762	0.217	0.021	*Oleriina*
Napeogenes	0.261	0.649	0.090	*Napeogenes*
Napeogenes	0.717	0.237	0.046	*Oleriina*
Napeogenes	0.309	0.656	0.035	*Napeogenes*
Napeogenes	0.169	0.426	0.405	*Hypothyris*
Napeogenes	0.754	0.231	0.014	*Oleriina*
Napeogenes	0.266	0.688	0.046	*Napeogenes*
Napeogenes	0.487	0.442	0.071	*Oleriina*
Napeogenes	0.555	0.346	0.098	*Oleriina*
Napeogenes	0.292	0.309	0.399	*Hypothyris*
Napeogenes	0.027	0.772	0.202	*Napeogenes*
Napeogenes	0.018	0.805	0.177	*Napeogenes*
Napeogenes	0.243	0.503	0.254	*Napeogenes*
Napeogenes	0.085	0.181	0.734	*Hypothyris*
Oleriina	0.669	0.219	0.112	*Oleriina*
Oleriina	0.347	0.540	0.113	*Napeogenes*
Oleriina	0.801	0.095	0.104	*Oleriina*
Oleriina	0.646	0.167	0.187	*Oleriina*
Oleriina	0.393	0.570	0.038	*Napeogenes*
Oleriina	0.498	0.472	0.030	*Oleriina*
Oleriina	0.614	0.164	0.222	*Oleriina*
Oleriina	0.900	0.025	0.075	*Oleriina*
Oleriina	0.734	0.143	0.123	*Oleriina*
Oleriina	0.553	0.130	0.318	*Oleriina*
Oleriina	0.398	0.273	0.329	*Oleriina*
Oleriina	0.599	0.060	0.341	*Oleriina*
Oleriina	0.814	0.080	0.107	*Oleriina*
Oleriina	0.661	0.175	0.164	*Oleriina*
Oleriina	0.786	0.102	0.112	*Oleriina*
Oleriina	0.276	0.473	0.252	*Napeogenes*
Oleriina	0.704	0.283	0.013	*Oleriina*
Oleriina	0.525	0.312	0.162	*Oleriina*
Oleriina	0.461	0.389	0.150	*Oleriina*
Oleriina	0.699	0.236	0.065	*Oleriina*
Oleriina	0.235	0.721	0.044	*Napeogenes*
Oleriina	0.327	0.228	0.445	*Hypothyris*
Oleriina	0.417	0.419	0.164	*Napeogenes*
Oleriina	0.907	0.079	0.014	*Oleriina*
Oleriina	0.944	0.035	0.022	*Oleriina*
Oleriina	0.255	0.600	0.145	*Napeogenes*
Oleriina	0.259	0.644	0.096	*Napeogenes*
Oleriina	0.430	0.318	0.252	*Oleriina*
Oleriina	0.335	0.582	0.083	*Napeogenes*
Oleriina	0.512	0.306	0.183	*Oleriina*
Oleriina	0.458	0.275	0.268	*Oleriina*
Oleriina	0.593	0.174	0.232	*Oleriina*
Oleriina	0.380	0.509	0.111	*Napeogenes*
Oleriina	0.607	0.184	0.208	*Oleriina*
Oleriina	0.605	0.293	0.102	*Oleriina*
Oleriina	0.542	0.047	0.411	*Oleriina*
Oleriina	0.490	0.087	0.423	*Hypothyris*
Oleriina	0.251	0.284	0.466	*Hypothyris*
Oleriina	0.650	0.079	0.271	*Oleriina*
Oleriina	0.424	0.093	0.483	*Hypothyris*
Oleriina	0.440	0.037	0.523	*Hypothyris*
Oleriina	0.521	0.162	0.318	*Oleriina*
Oleriina	0.276	0.086	0.638	*Hypothyris*
Oleriina	0.238	0.217	0.546	*Hypothyris*
Oleriina	0.264	0.078	0.658	*Hypothyris*
Oleriina	0.375	0.148	0.478	*Hypothyris*
Oleriina	0.418	0.244	0.338	*Oleriina*
Oleriina	0.465	0.045	0.490	*Hypothyris*
Oleriina	0.673	0.024	0.303	*Oleriina*
Oleriina	0.513	0.071	0.417	*Oleriina*
Oleriina	0.758	0.090	0.152	*Oleriina*
Oleriina	0.400	0.449	0.151	*Napeogenes*
Oleriina	0.628	0.181	0.191	*Oleriina*
Oleriina	0.731	0.091	0.178	*Oleriina*
Oleriina	0.070	0.860	0.070	*Napeogenes*
Oleriina	0.643	0.134	0.223	*Oleriina*
Oleriina	0.740	0.090	0.170	*Oleriina*
Oleriina	0.590	0.231	0.179	*Oleriina*
Oleriina	0.348	0.318	0.334	*Oleriina*
Oleriina	0.567	0.045	0.388	*Oleriina*
Oleriina	0.249	0.237	0.514	*Hypothyris*
Hypothyris	0.065	0.148	0.787	*Hypothyris*
Hypothyris	0.041	0.046	0.912	*Hypothyris*
Hypothyris	0.240	0.435	0.325	*Napeogenes*
Hypothyris	0.100	0.321	0.579	*Hypothyris*
Hypothyris	0.138	0.380	0.481	*Hypothyris*
Hypothyris	0.112	0.130	0.758	*Hypothyris*
Hypothyris	0.088	0.394	0.517	*Hypothyris*
Hypothyris	0.079	0.098	0.823	*Hypothyris*
Hypothyris	0.085	0.090	0.825	*Hypothyris*
Hypothyris	0.435	0.310	0.256	*Hypothyris*
Hypothyris	0.162	0.077	0.761	*Oleriina*
Hypothyris	0.073	0.033	0.894	*Hypothyris*
Hypothyris	0.223	0.318	0.458	*Hypothyris*
Hypothyris	0.062	0.020	0.918	*Hypothyris*
Hypothyris	0.058	0.097	0.845	*Hypothyris*
Hypothyris	0.059	0.180	0.761	*Hypothyris*
Hypothyris	0.305	0.270	0.425	*Hypothyris*
Hypothyris	0.119	0.110	0.771	*Hypothyris*
Hypothyris	0.385	0.097	0.518	*Hypothyris*
Hypothyris	0.149	0.036	0.815	*Hypothyris*
Hypothyris	0.217	0.118	0.665	*Hypothyris*
Hypothyris	0.143	0.164	0.692	*Hypothyris*
Hypothyris	0.115	0.022	0.863	*Hypothyris*
Hypothyris	0.123	0.176	0.701	*Hypothyris*
Hypothyris	0.026	0.004	0.970	*Hypothyris*
Hypothyris	0.367	0.244	0.389	*Hypothyris*
Hypothyris	0.022	0.076	0.902	*Hypothyris*
Hypothyris	0.732	0.183	0.085	*Hypothyris*
Hypothyris	0.127	0.061	0.812	*Oleriina*
Hypothyris	0.158	0.024	0.818	*Hypothyris*
Hypothyris	0.142	0.229	0.629	*Hypothyris*
Hypothyris	0.692	0.103	0.205	*Hypothyris*
Hypothyris	0.102	0.051	0.847	*Oleriina*
Hypothyris	0.085	0.068	0.847	*Hypothyris*
Hypothyris	0.359	0.341	0.300	*Hypothyris*
Hypothyris	0.581	0.291	0.128	*Oleriina*
Hypothyris	0.240	0.056	0.704	*Oleriina*
Hypothyris	0.389	0.183	0.428	*Hypothyris*
Hypothyris	0.284	0.223	0.494	*Hypothyris*
Hypothyris	0.132	0.109	0.759	*Hypothyris*
Hypothyris	0.461	0.062	0.477	*Hypothyris*
Hypothyris	0.346	0.069	0.584	*Hypothyris*
Hypothyris	0.465	0.242	0.292	*Hypothyris*
Hypothyris	0.198	0.208	0.594	*Oleriina*
Hypothyris	0.185	0.264	0.550	*Hypothyris*
Hypothyris	0.176	0.073	0.751	*Hypothyris*
Hypothyris	0.514	0.073	0.414	*Hypothyris*
Hypothyris	0.083	0.569	0.348	*Oleriina*
Hypothyris	0.307	0.314	0.379	*Napeogenes*
Hypothyris	0.693	0.200	0.106	*Napeogenes*
Hypothyris	0.629	0.200	0.171	*Oleriina*
Hypothyris	0.310	0.036	0.654	*Oleriina*
Hypothyris	0.298	0.562	0.141	*Napeogenes*
Hypothyris	0.457	0.126	0.417	*Napeogenes*
Hypothyris	0.644	0.245	0.110	*Oleriina*
Hypothyris	0.810	0.169	0.021	*Oleriina*
Hypothyris	0.102	0.201	0.697	*Oleriina*
Hypothyris	0.066	0.068	0.866	*Hypothyris*
Hypothyris	0.031	0.364	0.605	*Hypothyris*
Hypothyris	0.062	0.807	0.131	*Napeogenes*

% Correct

Oleriina	*Napeogenes*	*Hypothyris*
63.934	50.000	70.000

Table S6: MCMCglmm Results

Key
P < 0.05
P < 0.01
P < 0.001

A) Flight height (Neuropil ~ rCBR + Flight Height + (1|Species) + (1|Sex) + inverse matrix of phylogeny)

Neuropil	Post-mean	l-95% CI	u-95% CI	eff. Sample	pMCMC
ME	0.046	0.007	0.085	10000.000	0.019
LOP	0.042	0.000	0.083	10292.000	0.051
LOB	0.041	0.001	0.079	9609.000	0.041
aME	0.012	-0.047	0.069	10000.000	0.666
vLOB	-1.018	-1.721	-0.325	10000.000	0.004
AL	0.027	0.005	0.076	10000.000	0.025
AOTU	0.039	0.002	0.071	10000.000	0.023
OL	0.045	0.009	0.084	10330.000	0.018

B) Mimicry (Neuropil ~ rCBR + Mimicry pattern + (1|Species) + (1|Sex) + inverse matrix of phylogeny)

Neuropil	Post-mean	l-95% CI	u-95% CI	eff. Sample	pMCMC
ME	-0.021	-0.075	0.037	10000.000	0.430
LOP	-0.037	-0.092	0.023	10000.000	0.196
LOB	-0.032	-0.087	0.020	10000.000	0.225
aME	0.009	-0.068	0.080	10000.000	0.748
vLOB	-0.371	-1.416	0.732	10000.000	0.482
AL	-0.019	-0.067	0.029	10000.000	0.415
AOTU	-0.018	-0.068	0.027	10000.000	0.434
OL	-0.023	-0.076	0.032	10392.000	0.378

C) Sex (Neuropil ~ rCBR + Sex + (1|Species) + inverse matrix of phylogeny)

Neuropil	Post-mean	l-95% CI	u-95% CI	eff. Sample	pMCMC
ME	-0.021	-0.039	-0.004	10000.000	0.019
LOP	-0.015	-0.037	0.008	10000.000	0.201
LOB	-0.009	-0.028	0.010	10291.000	0.349
aME	-0.007	-0.043	0.027	10000.000	0.691
vLOB	1.275	0.813	1.740	10000.000	<0.001
AL	0.022	0.001	0.044	10000.000	0.044
AOTU	-0.062	-0.087	-0.036	10733.000	<0.001
OL	-0.020	-0.037	-0.003	8912.000	0.025

D) Species (Neuropil ~ rCBR + Species + (1|Sex) + inverse matrix of phylogeny

ME

Fixed effects	Post-mean	l-95% CI	u-95%	eff. Sample	pMCMC
Intercept	2.745	-15.556	15.482	10987.000	0.200
rCBR	0.638	0.548	0.728	10000.000	<0.001
Species - *Hyposcada_illinissa*	-0.054	-0.101	-0.008	10000.000	0.022
Species - *Hyposcada_kena*	-0.041	-0.126	0.040	10000.000	0.344
Species - *Hypothyris_anastasia*	0.030	-0.007	0.067	9333.000	0.115
Species - *Hypothyris_fluonia*	0.024	-0.014	0.065	10000.000	0.241
Species - *Hypothyris_mamercus*	0.047	-0.017	0.105	10000.000	0.129
Species - *Hypothyris_moebiusi*	-0.074	-0.123	-0.025	10000.000	0.002
Species - *Napeogenes_inachia*	-0.030	-0.084	0.023	10000.000	0.274
Species - *Napeogenes_larina*	-0.014	-0.053	0.027	10258.000	0.497
Species - *Napeogenes_pharo*	-0.001	-0.056	0.051	10000.000	0.959
Species - *Napeogenes_rhezia*	-0.069	-0.121	-0.019	10000.000	0.007
Species - *Napeogenes_sylphis*	-0.104	-0.148	-0.058	10000.000	<0.001
Species - *Oleria_assimilis*	-0.119	-0.206	-0.032	8818.000	0.006
Species - *Oleria_gunilla*	-0.099	-0.154	-0.048	10000.000	<0.001
Species - *Oleria_onega*	-0.079	-0.119	-0.040	10000.000	<0.001
Species - *Oleria_sexmaculata*	-0.079	-0.130	-0.032	10000.000	0.002
Random effects					
Sex	435.300	<0.001	867.800	5532.000	
Residuals					
	0.003	0.002	0.004	10000.000	
DIC - *with species*	-494.349				
DIC - *without species*	-432.604				
ΔDIC	61.745				

LOP

Fixed effects	Post-mean	l-95% CI	u-95%	eff. Sample	pMCMC
Intercept	2.760	-14.812	18.455	9921.000	0.213
rCBR	0.508	0.387	0.628	10000.000	<0.001
Species - *Hyposcada_illinissa*	-0.041	-0.106	0.019	10000.000	0.199
Species - *Hyposcada_kena*	-0.046	-0.157	0.066	10000.000	0.420
Species - *Hypothyris_anastasia*	0.061	0.012	0.111	10000.000	0.015
Species - *Hypothyris_fluonia*	0.005	-0.047	0.059	10000.000	0.835
Species - *Hypothyris_mamercus*	0.050	-0.032	0.132	10000.000	0.231
Species - *Hypothyris_moebiusi*	-0.080	-0.146	-0.014	10000.000	0.020
Species - *Napeogenes_inachia*	-0.060	-0.131	0.014	10356.000	0.105
Species - *Napeogenes_larina*	-0.019	-0.072	0.033	10555.000	0.491
Species - *Napeogenes_pharo*	-0.002	-0.073	0.068	10000.000	0.960
Species - *Napeogenes_rhezia*	-0.028	-0.096	0.041	11780.000	0.408
Species - *Napeogenes_sylphis*	-0.080	-0.143	-0.019	10000.000	0.010
Species - *Oleria_assimilis*	-0.088	-0.206	0.028	10000.000	0.135
Species - *Oleria_gunilla*	-0.115	-0.185	-0.043	10198.000	0.001
Species - *Oleria_onega*	-0.078	-0.132	-0.025	10000.000	0.004
Species - *Oleria_sexmaculata*	-0.124	-0.191	-0.058	10000.000	0.000
Random effects					
Sex	506.200	<0.001	937.900	7523.000	
Residuals					
	0.005	0.004	0.006	9839.000	
DIC - *with species*	-394.634				
DIC - *without species*	-361.456				
ΔDIC	33.178				

LOB

Fixed effects	Post-mean	l-95% CI	u-95%	eff. Sample	pMCMC
Intercept	2.065	-10.145	16.784	14020.000	0.206
rCBR	0.615	0.513	0.714	9688.000	<0.001
Species - *Hyposcada_illinissa*	-0.099	-0.150	-0.047	10000.000	<0.001
Species - *Hyposcada_kena*	-0.065	-0.155	0.026	10000.000	0.169
Species - *Hypothyris_anastasia*	-0.001	-0.043	0.039	10000.000	0.943
Species - *Hypothyris_fluonia*	-0.042	-0.086	0.001	10000.000	0.056
Species - *Hypothyris_mamercus*	-0.051	-0.117	0.019	10000.000	0.145
Species - *Hypothyris_moebiusi*	-0.109	-0.165	-0.057	10000.000	<0.001
Species - *Napeogenes_inachia*	-0.087	-0.142	-0.024	10000.000	0.004
Species - *Napeogenes_larina*	-0.085	-0.131	-0.043	10000.000	<0.001
Species - *Napeogenes_pharo*	-0.066	-0.124	-0.006	10000.000	0.028
Species - *Napeogenes_rhezia*	-0.110	-0.166	-0.057	10000.000	<0.001
Species - *Napeogenes_sylphis*	-0.143	-0.192	-0.088	11481.000	<0.001
Species - *Oleria_assimilis*	-0.184	-0.278	-0.083	10000.000	<0.001
Species - *Oleria_gunilla*	-0.155	-0.217	-0.098	10000.000	<0.001
Species - *Oleria_onega*	-0.101	-0.146	-0.058	10000.000	<0.001
Species - *Oleria_sexmaculata*	-0.127	-0.182	-0.073	10000.000	<0.001
Random effects					
Sex	410.700	<0.001	621.200	7515.000	
Residuals					
	0.004	0.003	0.004	10000.000	
DIC - *with species*	-459.979				
DIC - *without species*	-418.069				
ΔDIC	41.911				

aME

Fixed effects	Post-mean	l-95% CI	u-95%	eff. Sample	pMCMC
Intercept	-1.326	-18.022	12.801	13927.000	0.365
rCBR	0.775	0.588	0.966	10000.000	<0.001
Species - *Hyposcada_illinissa*	0.100	0.005	0.197	10000.000	0.038
Species - *Hyposcada_kena*	-0.197	-0.371	-0.027	10345.000	0.025
Species - *Hypothyris_anastasia*	0.070	-0.008	0.145	10000.000	0.073
Species - *Hypothyris_fluonia*	0.009	-0.073	0.094	10234.000	0.830
Species - *Hypothyris_mamercus*	0.017	-0.111	0.139	8820.000	0.799
Species - *Hypothyris_moebiusi*	0.040	-0.058	0.143	10000.000	0.423
Species - *Napeogenes_inachia*	0.004	-0.105	0.117	11314.000	0.943
Species - *Napeogenes_larina*	0.066	-0.017	0.149	10000.000	0.118
Species - *Napeogenes_pharo*	0.148	0.039	0.255	10000.000	0.009
Species - *Napeogenes_rhezia*	0.063	-0.048	0.170	10000.000	0.261
Species - *Napeogenes_sylphis*	0.090	-0.007	0.187	10000.000	0.067
Species - *Oleria_assimilis*	0.116	-0.065	0.304	10000.000	0.226
Species - *Oleria_gunilla*	0.051	-0.062	0.161	10000.000	0.365
Species - *Oleria_onega*	-0.005	-0.086	0.077	10522.000	0.893
Species - *Oleria_sexmaculata*	-0.033	-0.136	0.070	10000.000	0.527
Random effects					
Sex	1116.000	<0.001	829.800	10000.000	
Residuals					
	0.012	0.010	0.015	10000.000	
DIC - *with species*	-246.132				
DIC - *without species*	-241.787				
ΔDIC	4.345				

vLOB

Fixed effects	Post-mean	l-95% CI	u-95%	eff. Sample	pMCMC
Intercept	-8.989	-40.881	24.298	10000.000	0.478
rCBR	1.680	-0.833	4.299	10000.000	0.207
Species - *Hyposcada_illinissa*	-0.079	-1.250	1.227	10000.000	0.900
Species - *Hyposcada_kena*	0.011	-2.240	2.187	10000.000	0.994
Species - *Hypothyris_anastasia*	-1.093	-2.134	-0.117	10000.000	0.034
Species - *Hypothyris_fluonia*	-0.658	-1.785	0.401	10000.000	0.239
Species - *Hypothyris_mamercus*	-2.408	-4.045	-0.709	9707.000	0.004
Species - *Hypothyris_moebiusi*	-1.436	-2.784	-0.145	10000.000	0.035
Species - *Napeogenes_inachia*	-2.865	-4.580	-1.240	10000.000	0.001
Species - *Napeogenes_larina*	-1.808	-2.920	-0.676	10404.000	0.001
Species - *Napeogenes_pharo*	-1.960	-3.360	-0.536	10952.000	0.007
Species - *Napeogenes_rhezia*	0.147	-1.278	1.537	10000.000	0.846
Species - *Napeogenes_sylphis*	-1.501	-2.804	-0.114	10000.000	0.030
Species - *Oleria_assimilis*	-1.134	-3.588	1.283	10000.000	0.360
Species - *Oleria_gunilla*	0.107	-1.400	1.580	10000.000	0.891
Species - *Oleria_onega*	-0.042	-1.133	1.064	10000.000	0.937
Species - *Oleria_sexmaculata*	-0.306	-1.648	1.009	10241.000	0.651
Random effects					
Sex	864.900	0.047	2167.000	9718.000	
Residuals					
	2.086	1.636	2.597	10000.000	
DIC - *with species*	582.252				
DIC - *without species*	596.471				
ΔDIC	14.219				

AL

Fixed effects	Post-mean	l-95% CI	u-95%	eff. Sample	pMCMC
Intercept	-0.038	-16.440	16.504	8658.000	0.945
rCBR	0.884	0.771	0.998	10000.000	<0.001
Species - *Hyposcada_illinissa*	-0.058	-0.115	0.003	10000.000	0.051
Species - *Hyposcada_kena*	-0.104	-0.204	0.004	10000.000	0.053
Species - *Hypothyris_anastasia*	0.031	-0.015	0.080	9715.000	0.191
Species - *Hypothyris_fluonia*	-0.028	-0.076	0.024	10000.000	0.282
Species - *Hypothyris_mamercus*	0.062	-0.013	0.140	10000.000	0.112
Species - *Hypothyris_moebiusi*	-0.030	-0.088	0.036	10415.000	0.343
Species - *Napeogenes_inachia*	0.053	-0.018	0.120	10000.000	0.134
Species - *Napeogenes_larina*	0.040	-0.011	0.090	10000.000	0.115
Species - *Napeogenes_pharo*	0.068	0.001	0.136	10854.000	0.046
Species - *Napeogenes_rhezia*	0.044	-0.022	0.105	10000.000	0.185
Species - *Napeogenes_sylphis*	0.014	-0.047	0.072	10000.000	0.649
Species - *Oleria_assimilis*	-0.030	-0.139	0.085	10000.000	0.591
Species - *Oleria_gunilla*	-0.046	-0.111	0.021	10000.000	0.176
Species - *Oleria_onega*	-0.034	-0.085	0.017	10000.000	0.194
Species - *Oleria_sexmaculata*	-0.052	-0.111	0.011	9690.000	0.102
Random effects					
Sex	851.600	<0.001	839.300	10000.000	
Residuals					
	0.005	0.004	0.006	9982.000	
DIC - *with species*	-414.049				
DIC - *without species*	-395.917				
ΔDIC	18.133				

AOTU

Fixed effects	Post-mean	l-95% CI	u-95%	eff. Sample	pMCMC
Intercept	-0.023	-0.196	2.040	8918.000	0.841
rCBR	0.735	0.600	0.870	9713.000	<0.001
Species - *Hyposcada_illinissa*	-0.071	-0.140	-0.002	10003.000	0.041
Species - *Hyposcada_kena*	0.033	-0.095	0.154	10000.000	0.610
Species - *Hypothyris_anastasia*	0.054	-0.002	0.110	10341.000	0.062
Species - *Hypothyris_fluonia*	0.031	-0.029	0.091	10338.000	0.288
Species - *Hypothyris_mamercus*	0.161	0.065	0.248	11168.000	0.001
Species - *Hypothyris_moebiusi*	0.003	-0.007	0.078	10000.000	0.939
Species - *Napeogenes_inachia*	0.051	-0.033	0.129	10675.000	0.214
Species - *Napeogenes_larina*	0.013	-0.046	0.075	10000.000	0.668
Species - *Napeogenes_pharo*	0.039	-0.040	0.119	10000.000	0.342
Species - *Napeogenes_rhezia*	<0.001	-0.078	0.074	10000.000	0.991
Species - *Napeogenes_sylphis*	-0.004	-0.109	0.030	10000.000	0.294
Species - *Oleria_assimilis*	-0.035	-0.167	0.097	10142.000	0.603
Species - *Oleria_gunilla*	-0.002	-0.084	0.074	10000.000	0.950
Species - *Oleria_onega*	-0.014	-0.076	0.044	10000.000	0.632
Species - *Oleria_sexmaculata*	0.018	0.054	0.094	10000.000	0.627
Random effects					
Sex	832.200	<0.001	1201.000	6411.000	
Residuals					
	0.007	0.005	0.008	10000.000	
DIC - *with species*	-354.683				
DIC - *without species*	-346.533				
ΔDIC	8.149				

OL

Fixed effects	Post-mean	l-95% CI	u-95%	eff. Sample	pMCMC
Intercept	3.137	-12.893	19.060	12817.000	0.204
rCBR	0.630	0.539	0.719	10000.000	<0.001
Species - *Hyposcada_illinissa*	-0.058	-0.104	-0.014	9640.000	0.012
Species - *Hyposcada_kena*	-0.044	-0.124	0.040	10000.000	0.286
Species - *Hypothyris_anastasia*	0.028	-0.008	0.065	10000.000	0.128
Species - *Hypothyris_fluonia*	0.016	-0.023	0.053	10000.000	0.424
Species - *Hypothyris_mamercus*	0.036	-0.025	0.096	10000.000	0.237
Species - *Hypothyris_moebiusi*	-0.078	-0.125	-0.028	10707.000	0.001
Species - *Napeogenes_inachia*	-0.037	-0.090	0.016	10000.000	0.170
Species - *Napeogenes_larina*	-0.022	-0.061	0.018	10000.000	0.278
Species - *Napeogenes_pharo*	-0.008	-0.058	0.046	10290.000	0.763
Species - *Napeogenes_rhezia*	-0.071	-0.121	-0.023	10000.000	0.006
Species - *Napeogenes_sylphis*	-0.107	-0.152	-0.061	10000.000	<0.001
Species - *Oleria_assimilis*	-0.124	-0.211	-0.038	10000.000	0.005
Species - *Oleria_gunilla*	-0.105	-0.155	-0.053	11053.000	<0.001
Species - *Oleria_onega*	-0.081	-0.121	-0.044	10382.000	<0.001
Species - *Oleria_sexmaculata*	-0.086	-0.136	-0.039	10294.000	0.001
Random effects					
Sex	379.900	<0.001	927.400	3738.000	
Residuals					
	0.003	0.002	0.003	10000.000	
DIC - *with species*	-500.527				
DIC - *without species*	-439.187				
ΔDIC	61.340				

UOB Open
